# Alveolar–Capillary Barrier Protection In Vitro: Lung Cell Type-Specific Effects and Molecular Mechanisms Induced by 1α, 25-Dihydroxyvitamin D3

**DOI:** 10.3390/ijms24087298

**Published:** 2023-04-14

**Authors:** Junyu Xiong, Patrick Kaleja, Larissa Ückert, Niloufar Nezaratizadeh, Stefanie Krantz, Martin Friedrich Krause, Stefanie Fitschen-Oestern, Andreas Seekamp, Liam Cassidy, Andreas Tholey, Sabine Fuchs

**Affiliations:** 1 Experimental Trauma Surgery, Department of Trauma Surgery and Orthopedics, University Medical Center Schleswig-Holstein, Campus Kiel, 24105 Kiel, Germany; 2 Systematic Proteomics & Bioanalytics, Institute for Experimental Medicine, Christian-Albrechts-Universität zu Kiel, 24015 Kiel, Germany; 3 Department of Pediatrics, University Medical Center Schleswig-Holstein, Campus Kiel, 24105 Kiel, Germany

**Keywords:** VD3, lung barrier, alveolar epithelial cells, co-culture, LPS, DCLK1, LC-MS, quantitative proteomics

## Abstract

Low serum levels of 1α, 25-dihydroxyvitamin D3 (VD3) are associated with a higher mortality in trauma patients with sepsis or ARDS. However, the molecular mechanisms behind this observation are not yet understood. VD_3_ is known to stimulate lung maturity, alveolar type II cell differentiation, or pulmonary surfactant synthesis and guides epithelial defense during infection. In this study, we investigated the impact of VD_3_ on the alveolar–capillary barrier in a co-culture model of alveolar epithelial cells and microvascular endothelial cells respectively in the individual cell types. After stimulation with bacterial LPS (lipopolysaccharide), gene expression of inflammatory cytokines, surfactant proteins, transport proteins, antimicrobial peptide, and *doublecortin-like kinase 1 (DCLK1)* were analyzed by real-time PCR, while corresponding proteins were evaluated by ELISA, immune-fluorescence, or Western blot. The effect of VD3 on the intracellular protein composition in H441 cells was analyzed by quantitative liquid chromatography-mass spectrometry-based proteomics. VD_3_ effectively protected the alveolar–capillary barrier against LPS treatment, as indicated by TEER measurement and morphological assessment. VD_3_ did not inhibit the IL-6 secretion by H441 and OEC but restricted the diffusion of IL-6 to the epithelial compartment. Further, VD_3_ could significantly suppress the surfactant protein A expression induced in the co-culture system by LPS treatment. VD_3_ induced high levels of the antimicrobial peptide LL-37, which counteracted effects by LPS and strengthened the barrier. Quantitative proteomics identified VD3-dependent protein abundance changes ranging from constitutional extracellular matrix components and surfactant-associated proteins to immune-regulatory molecules. DCLK1, as a newly described target molecule for VD_3_, was prominently stimulated by VD_3_ (10 nM) and seems to influence the alveolar–epithelial cell barrier and regeneration.

## 1. Introduction

The alveolar epithelium and the circumferential capillary endothelium in the lung constitute the alveolar–capillary barrier, serving as a functional unit to regulate gas exchange during respiration [1] This barrier is highly sensible due to several reasons including the large surface area of the alveolar region, its low thickness, and the multiple factors affecting the barrier such as infection, inflammation, and mechanical stress. Impairment of the barrier function in clinical syndromes such as acute lung injury or ARDS still resembles a severe clinical challenge and is associated with a high morbidity and mortality for critically ill patients.

The active form of vitamin D3, 1α, 25-dihydroxyvitamin D_3_ (VD_3_), has been reported to maintain the integrity of different tissues and biological barriers, e.g., intestine, cornea, or brain [2,3,4,5]. Furthermore, VD_3_ also plays a role in lung development and maturation guiding lung cell proliferation and differentiation [6,7,8] and has been shown to effect the function of alveolar type II (ATII) cells mediating the repair of the epithelial–alveolar barrier [9,10]. Human serum levels of VD_3_ around 50 nM (20 ng/mL) are recommended [11], but active VD3 can be generated and stored in the lung tissue, emphasizing the importance of VD3 for locally mediated processes in the lung. On the other hand, when the VD_3_ levels in the blood serum are reduced, people have a higher risk to suffer from acute lung injury/acute respiratory distress syndrome (ALI/ARDS), asthma, and chronic respiratory diseases [12,13,14,15,16,17,18]. Furthermore, it has been demonstrated that vitamin D/VDR signaling has a direct influence on the maintenance of the integrity of the pulmonary epithelial barrier and effectively alleviates lung injury induced by the bacterial endotoxin, lipopolysaccharide (LPS), and treatment [19]. Nevertheless, due to the complexity of VD3-triggered processes in the lung, the detailed mechanisms supporting the barrier function are not fully understood. In addition, the identification of VD3-responsive target molecules involved in maintaining or restoring the alveolar–capillary barrier of the lung might lead to new concepts to treat lung diseases such as ALI/ARDS or support other clinical measures in the case of ALI/ARDS.

On the molecular level, the biological functions of VD_3_ are mediated by vitamin D/vitamin D receptor (VDR) signaling pathways existing in a variety of cells including the lung epithelial cells [20,21]. VD_3_ is delivered into the cell after binding to vitamin D binding protein (VDBP), followed by binding to the VDR, a member of the nuclear receptors that is featured as a ligand-activated transcription factor [20,22,23]. After the translocation of vitamin D/VDR complex into the nucleus and dimerization with the retinoid-X receptor (RXR) [24], this heterodimer interacts with vitamin D response elements (VDRE) in the promoter regions [25] and regulates the gene expression associated with a wide spectrum of biological processes, such as the defense against cancer and infections [26,27], and the maintenance of tissue and organ functions [28].

In this study, we assessed cell type-specific effects and associated molecular mechanisms induced by active VD3 (1α, 25-dihydroxyvitamin D3) in the alveolar–capillary barrier using a co-culture model of alveolar epithelial NCI-H441 cells and microvascular endothelial cells (outgrowth endothelial cells, OECs) as well as mono-cultures of the individual cell types. The protective effect of VD3 was evaluated by challenging the system with IL-6 or LPS as key factors leading to the damage of the alveolar–capillary barrier in ALI or ARDS. The co-culture system on the Transwells^®^ allows a site-directed exposure in accordance with the two-sited alveolar–capillary interface, resulting in an epithelial and an endothelial compartment. In addition, this system supports assessing site- or cell type-specific effects in response to LPS or protective compounds, as published before [29,30].

The impact of VD_3_ on maintaining the barrier in response to LPS in the co-culture as a functional unit as well as in individual cell types was measured by transepithelial electrical resistance (TEER) or impedance measurement, respectively. In addition, we evaluated the influence of VD3 on cytokine profiles in the individual compartments and cell types, as well as associated immune regulatory molecules or antimicrobial molecules in response to VD3 in epithelial cells. To identify additional VD3-triggered target proteins in epithelial cells, a quantitative proteome analysis based on isobaric labeling and liquid chromatography-mass spectrometry (LC-MS) was performed. Selected molecules were further investigated on the cell and molecular levels with respect to their VD3-dependent expression or impact on barrier maintenance.

## 2. Results

**Figure 1:**
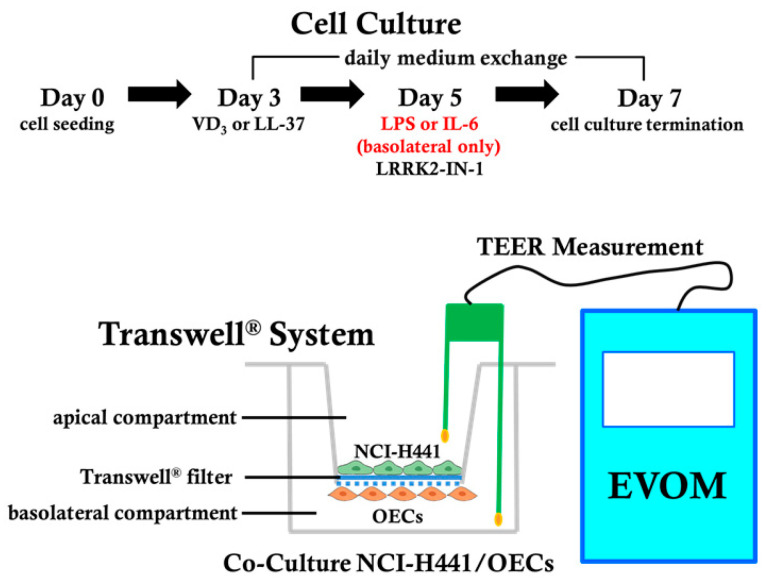
Experimental setup and design of the co-culture of NCI-H441/OECs in the Transwell^®^ systems for the TEER measurement.

The experimental setup for the co-culture model of epithelial (NCI-H441) and microvascular endothelial cells (OECs) on Transwells^®^ to simulate the human alveolar–capillary barrier is illustrated in Figure 1. On day 3, the cells were pretreated with VD_3_ (10 nM). The barrier was challenged using IL-6 (10 ng/mL) or LPS (100 ng/mL, 1 μg/mL, 10 μg/mL), and treatments were performed on day 5 solely to the basolateral compartments.

### 2.1. Effect of VD_3_ on the Barrier Integrity of the Alveolar–Capillary Interface In Vitro

**Figure 2:**
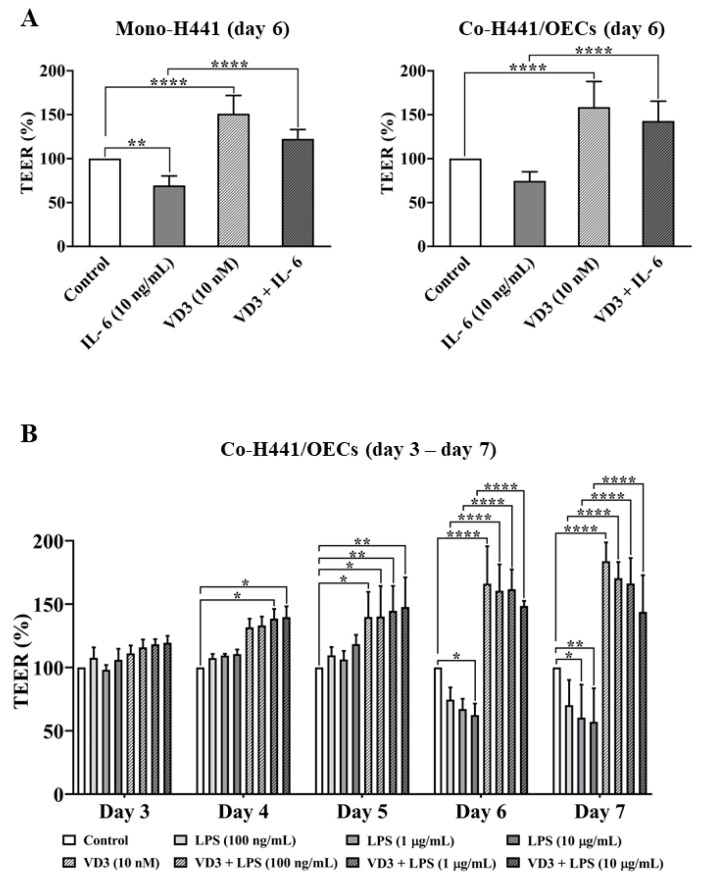
TEER measurement from the Transwell^®^ systems to investigate the effects of VD_3_ (10 nM) on the barrier integral resistance. (**A**) TEER measurement on day 6 of the mono-culture of NCI-H441 cells and co-culture of NCI-H441/OECs treated with VD_3_ (10 nM) and IL-6 (10 ng/mL). (**B**). Temporal development of TEER from day 3 to day 7 of the co-culture of NCI-H441/OECs treated with VD_3_ (10 nM) and different doses of LPS (100 ng/mL, 1 μg/mL, 10 μg/mL). The treatment was performed as depicted schematically in Figure 1 for IL-6 or LPS in independent experiments. The measured TEER values were analyzed as relative ratios in percentage (%) in comparison with the control group and shown as mean values ± SD from at least three independent experimental sets (*n* ≥ 3). * *p* ˂ 0.05, ** *p* ˂ 0.01, **** *p* ˂ 0.0001, two-way ANOVA.

The impact of VD_3_ on the barrier in response to IL-6 exposure was measured by TEER assessment in both mono-cultures of NCI-H441 cells and co-cultures of NCI-H441/OECs. TEER values are depicted as percentage in comparison to the control group. After the cells were pretreated with VD_3_ (10 nM) on day 3 and treated with IL-6 (10 ng/mL) from the basolateral side on day 5, the TEER values on day 6 were decreased in both mono-cultures and co-cultures. A more prominent reduction of TEER values was noticed in the mono-cultures of NCI-H441 cells (Figure 2A), implicating a detrimental effect of IL-6 on the epithelial barrier. However, VD_3_ (10 nM) showed a remarkable effect to increase the barrier integrity, showing increases of 51 ± 20.8% and 58.7 ± 29.07% in mono- and co-culture systems, respectively (Figure 2A). Furthermore, VD3 showed a noticeable effect to strengthen the barrier functionality in response to IL-6 exposure in mono- and co-culture systems (Figure 2A).

The impact of VD_3_ on the alveolar–capillary barrier in the co-culture systems was further investigated using different doses of LPS (100 ng/mL, 1 µg/mL, 10 µg/mL). NCI-H441 cells on the apical side and OECs on the basolateral side of the Transwells^®^ were pretreated with VD_3_ (10 nM) on day 3. From the basolateral side, LPS treatments were performed from day 5 to day 7 for 48 h. Similar to the results for IL-6, VD_3_ (10 nM) significantly improved the barrier resistance, whereas LPS showed a dose-dependent effect to decrease the barrier, as indicated by TEER values on day 6 and day 7 (Figure 2B). Nevertheless, the barrier integrity was preserved by VD_3_ (10 nM) when exposed to various concentrations of LPS (Figure 2B). These results indicate a prominent effect of VD_3_ (10 nM) on the alveolar–capillary barrier protection in the case of infection.

As TEER measurement by EVOM mainly indicates the barrier function provided by the epithelial cells, we additionally tested the impact of VD_3_ on endothelial cells using impedance measurement in an ECIS testing system. Although LPS lowered the barrier in endothelial cells and showed reduced impedance values, VD_3_ showed no protective effect in the endothelial cells (Supplemental Figure S1).

**Figure 3:**
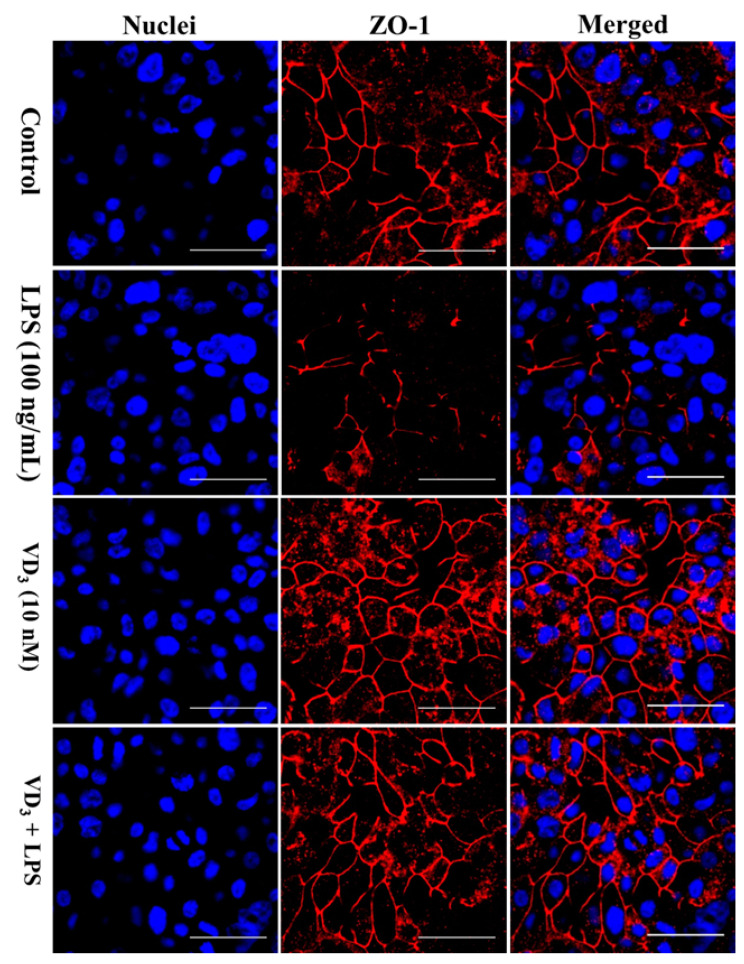
Immunofluorescence analysis of the tight junction molecule, ZO-1, in NCI-H441 cells. The NCI-H441 cells were treated with the conditioned media from the mono-culture of VD_3_ (10 nM) and LPS (100 ng/mL)-treated OECs to restore the co-culture condition in the Transwell^®^ system. The cell nuclei were counterstained with Hoechst (blue) and ZO-1 in NCI-H441 cells was stained with anti-ZO-1 antibody (rabbit) and Alexa 555 (donkey, anti-rabbit) (red), magnification: 63 folds, scale bar: 50 μm.

The protective effect of VD_3_ on the alveolar–epithelial barrier was also verified on the morphological level by the immunofluorescent staining for the tight junction protein ZO-1. The NCI-H441 cells were pretreated with VD_3_ and then incubated with conditioned medium derived from OECs exposed to LPS (100 ng/mL) for 48 h. The results exhibited a noticeable impairment of the tight junctional structure by the LPS (100 ng/mL), whereas the clear junctional structure along cell borders could be observed in VD_3_-pretreated NCI-H441 cells (Figure 3).

### 2.2. The Impact of VD_3_ on the Inflammatory Response in NCI-H441 Cells and OECs in Response to LPS Exposure

In our prior studies, we have shown that the endothelial-mediated inflammation caused by LPS is a major factor to damage the barrier and increase the barrier permeability in epithelial cells [29]. In virtue of the barrier impairment caused by the IL-6 (10 ng/mL) treatment (Figure 2A), we quantified the IL-6 levels in the co-culture of NCI-H441/OECs on the Transwells^®^ in response to the LPS and evaluated the impact of VD_3_.

**Figure 4:**
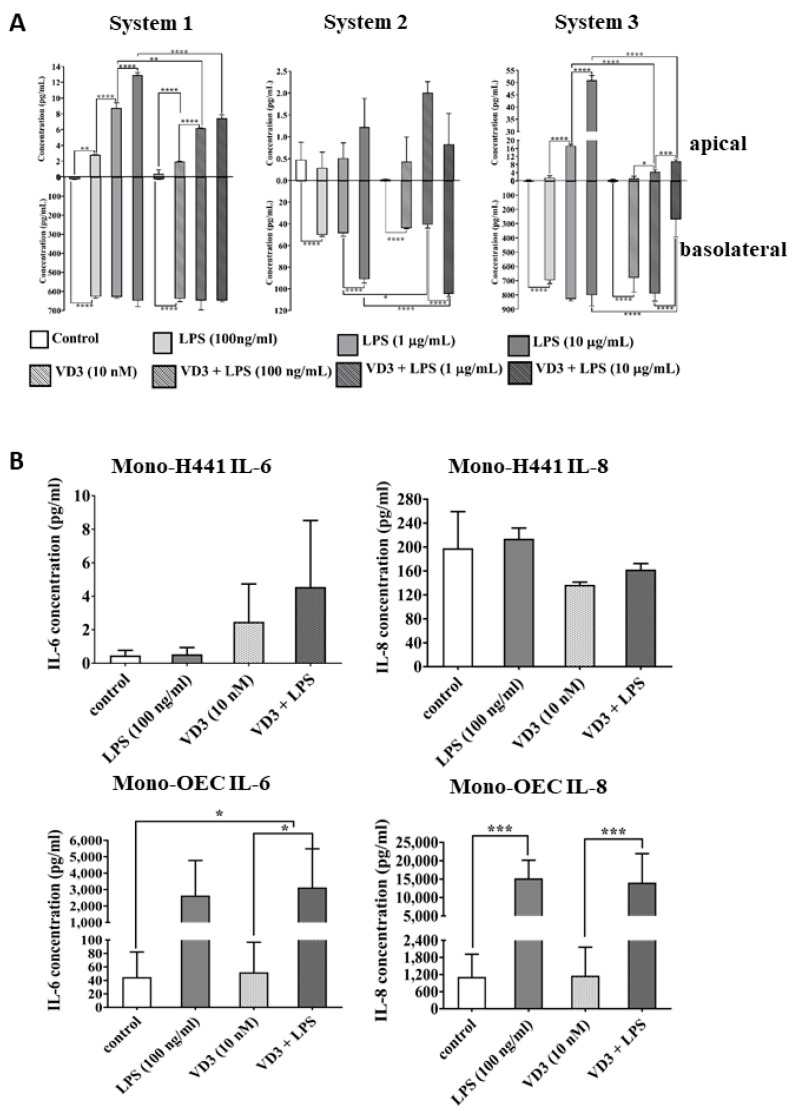
Secretion analyses of pro-inflammatory cytokines from NCI-H441 cells and OECs from different cell culture systems. (**A**) IL-6 secretion from the co-culture of NCI-H441/OECs treated with VD_3_ (10 nM) and different doses of LPS (100 ng/mL, 1 μg/mL, 10 μg/mL). The supernatants were collected on day 7 from both apical and basolateral compartments of Transwells^®^ from three individual co-culture experimental sets (*n* = 3). (**B**) IL-6 and IL-8 secretions from the mono-culture of NCI-H441 cells and OECs treated with VD_3_ (10 nM) and LPS (100 ng/mL). The supernatants were collected on day 7 from the mono-cultures. The values were depicted as pg/mL and shown as mean values ± SD from at least three independent experimental sets (*n* ≥ 3). * *p* ˂ 0.05, ** *p* ˂ 0.01, *** *p* ˂ 0.001, **** *p* ˂ 0.0001, one-way ANOVA.

The concentration of IL-6 in the apical compartments and in the basolateral compartment in the co-culture systems was analyzed by IL-6 ELISA. The results display the IL-6 concentrations in the individual co-culture systems of three replicates using the Transwells^®^. The IL-6 concentrations in both apical and basolateral compartments showed a dose-dependent correlation to LPS (100 ng/mL, 1 μg/mL, 10 μg/mL) treatments (Figure 4A). The IL-6 concentration levels in the basolateral compartments were substantially higher (max. to 828.2 ± 12.6 pg/mL) (Figure 4A) than in the apical compartments (max. to 50.93 ± 1.91 pg/mL) (Figure 4A) in response to LPS (100 ng/mL, 1 μg/mL, 10 μg/mL) treatments, which indicates a high inflammatory response of endothelial cells during the infection. VD_3_ (10 nM) showed a significant effect to decrease the IL-6 concentrations in the apical compartments when higher doses of LPS (1 μg/mL, 10 μg/mL) were applied (Figure 4A). However, the effect of VD_3_ (10 nM) in the basolateral compartments showed a high variation for each experimental set, which could be attributed to the different reactive patterns of OECs donors, and no clear trend for a protective role of VD3 on IL-6 in the basolateral compartment was noticeable in the donor datasets.

In addition, we analyzed the cytokine levels of IL-6 and IL-8 in the mono-culture of NCI-H441 cells and OECs to obtain deeper insight into the origin of the cytokines and to identify cell type-specific reaction patterns. Cytokines were monitored on the protein level (Figure 4B) and gene expression level (Supplemental Figure S2). The secretion levels of IL-6 and IL-8 in NCI-H441 cells were not significantly affected by VD_3_ (10 nM) and LPS (100 ng/mL), and relative low doses of IL-6 were noticed in NCI-H441 cells (Figure 4B). In contrast, the cytokines in OECs were highly raised when exposed to LPS (Figure 4B). However, VD_3_ did not show any obvious influence on IL-6 and IL-8 levels in OECs (Figure 4B). In addition, these results further confirmed that endothelial cells are the main source of inflammatory factors in the barrier in response to LPS, leading to epithelial barrier dysfunction. In the co-culture systems, however, VD_3_ (10 nM) treatment could remarkably reduce the cytokine levels in the epithelial compartment when exposed to higher doses of LPS (1 μg/mL, 10 μg/mL) (Figure 4A). This observation is possibly resulting from a stronger and less permeable epithelial barrier induced by the VD_3_ limiting free diffusion of inflammatory cytokines.

### 2.3. Effects of VD_3_ on Gene Expression of Epithelial Defense and Immune Regulators

**Figure 5:**
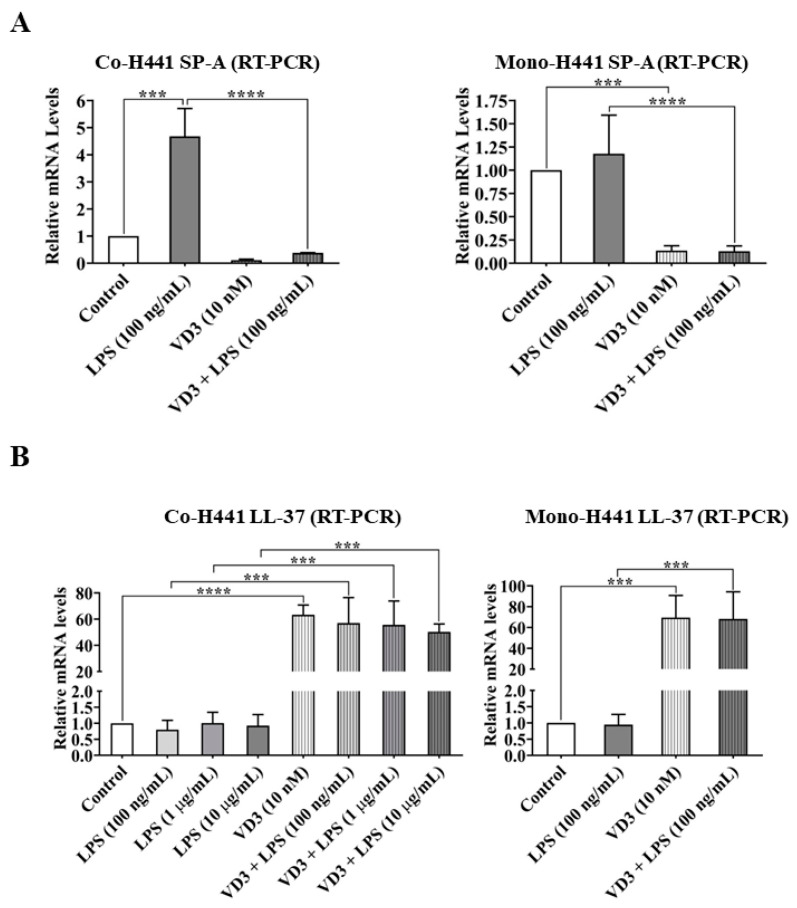
mRNA levels of SP-A and LL-37 in NCI-H441 cells. (**A**) mRNA levels of SP-A in NCI-H441 cells from the co-culture of NCI-H441/OECs and mono-culture of NCI-H441 cells treated with VD_3_ (10 nM) and LPS (100 ng/mL). (**B**) mRNA levels of LL-37 in NCI-H441 cells from both the co-culture of NCI-H441/OECs treated with VD_3_ (10 nM) and different doses of LPS (100 ng/mL, 1 μg/mL, 10 μg/mL) and the mono-culture of NCI-H441 cells treated with VD_3_ (10 nM) and LPS (100 ng/mL). The mRNA levels were analyzed through real-time PCR. The values were shown as mean values ± SD from at least three independent experiments (*n* ≥ 3). *** *p* ˂ 0.001, **** *p* ˂ 0.0001, one-way ANOVA.

The study also investigated the influence of VD_3_ on the gene expression of the surfactant protein SP-A as an important mediator of the innate immune system in alveolar epithelial cells. The gene expression of SP-A in NCI-H441 cells was significantly induced by the LPS (100 ng/mL) infection in the co-culture of NCI-H441/OECs, whereas no significant changes were observed in the mono-culture systems (Figure 5A). This result implicated the considerable effect of cellular crosstalk on the SP-A regulation, which could be attributed to pro-inflammatory cytokines, such as IL-6 and IL-8, produced from OECs. However, VD_3_ (10 nM) significantly suppressed the mRNA levels of SP-A in NCI-H441.

Besides SP-A, the expression of the antimicrobial peptide LL-37 in NCI-H441 cells was analyzed in response to VD_3_ in both co-cultures of NCI-H441/OECs and mono-cultures of NCI-H441 cells (Figure 5B). VD_3_ significantly induced the mRNA levels of LL-37 in NCI-H441 cells in both co- and mono-culture systems, whereas its gene expression was apparently not affected by LPS (Figure 5B).

### 2.4. VD_3_-Mediated Effect of Cathelicidin on Epithelial Barrier Protection

**Figure 6:**
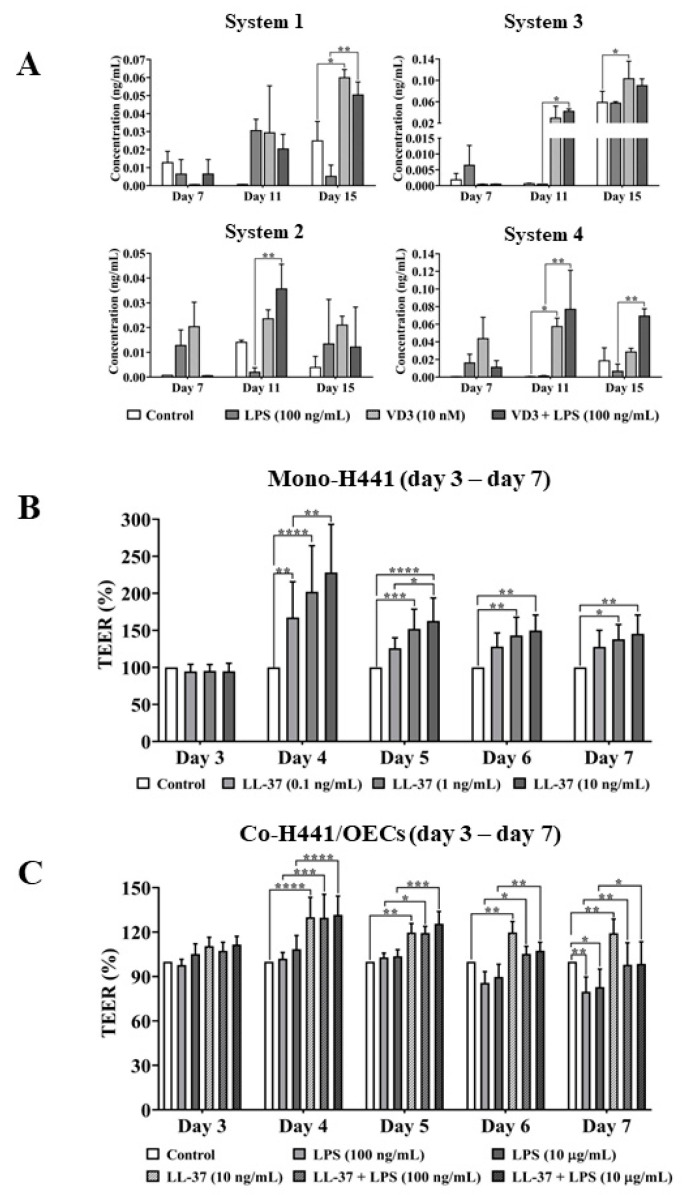
The secretion levels of LL-37 in NCI-H441 cells and its effect on the barrier properties. (**A**) LL-37 secretion of NCI-H441 cells from four individual modified mono-cultures of NCI-H441 cells with VD_3_ (10 nM) and LPS (100 ng/mL) treatments (*n* = 4). The cell culture period was extended to 15 days, and the medium supernatants were collected on day 7, 11, and 15, with medium exchange every 4 days. (**B**) TEER measurement from day 3 to day 7 of the mono-culture of NCI-H441 cells grown on the apical side of the Transwell^®^ filters. The 1 µM dexamethasone and LL-37 treatments were applied from day 3 to day 7 with different doses (0.1 ng/mL, 1 ng/mL, 10 ng/mL). (**C**) TEER measurement from day 3 to day 7 of the co-culture of NCI-H441/OECs in the Transwells^®^. The cells were pretreated with LL-37 (10 ng/mL) and 1 µM dexamethasone on day 3 and treated with LPS (100 ng/mL, 10 µg/mL) on day 5 from the basolateral compartments for 48 h. The measured TEER values were analyzed as relative ratios in percentage (%) in comparison with the control group. The values were shown as mean values ± SD from at least four independent experiments (*n* ≥ 4). * *p* ˂ 0.05, ** *p* ˂ 0.01, *** *p* ˂ 0.001, **** *p* ˂ 0.0001, one- and two-way ANOVA.

The secretion of LL-37 by NCI-H441 cells was further analyzed in slightly modified mono-culture systems, and the results are presented for four individual mono-culture sets (Figure 6A). The concentrations of secreted LL-37 reached up to 0.069 ± 0.008 ng/mL in the individual setups. However, the LL-37 production showed either an increasing trend or a significant increase in LL-37 in response to VD_3_. Nevertheless, the time frames for the impact of VD_3_ and LPS on LL-37 showed some variations in the individual sets, although experiments for individual sets were carried out in the same way and suggest the induction of LL-37 secretion in epithelial cells by VD_3_.

In consequence, we investigated the direct impact of LL-37 (10 nM) on the epithelial barrier properties (Figure 6B). First, different doses of LL-37 (0.1 ng/mL, 1 ng/mL, 10 ng/mL) were applied to the mono-culture of NCI-H441 cells cultured in the apical compartments of Transwells^®^ on day 3, and the barrier resistance was monitored from day 3 to day 7. The results exhibited a significant effect of LL-37 (0.1 ng/mL, 1 ng/mL, 10 ng/mL) to strengthen the barrier properties in a dose-dependent manner (Figure 6B), although the impact seemed to decrease with ongoing culture time. In addition, in the co-culture of NCI-H441/OECs, LL-37 (10 ng/mL) revealed a significant effect to increase the barrier properties, and the barrier integrity was remarkably maintained by LL-37 (10 ng/mL) in the context of exposure to LPS (100 ng/mL, 1 µg/mL) (Figure 6C).

### 2.5. Quantitative Proteomics Identifies VD_3_-Induced Changes in Protein Abundances in NCI-H441 Epithelial Cells

In order to elucidate molecular processes associated with VD_3_ treatment for the epithelial barrier and to identify potential target proteins of VD_3_ in NCI-H441 cells, VD_3_ (10 nM)-treated NCI-H441 cells were subjected to a quantitative proteome analysis employing a bottom-up approach. Protein identification and quantification was performed using isobaric labeling, employing the tandem mass tag (TMT) technology [31], followed by two-dimensional liquid chromatography-tandem mass spectrometry (LC-MS/MS). Three biological replicates of both control and VD_3_-treated samples were combined into a single TMT-sixplex experiment. From this, four technical replicates (LC-MS/MS) were performed, including an exclusion list to increase proteome coverage in the fourth replicate.

**Figure 7:**
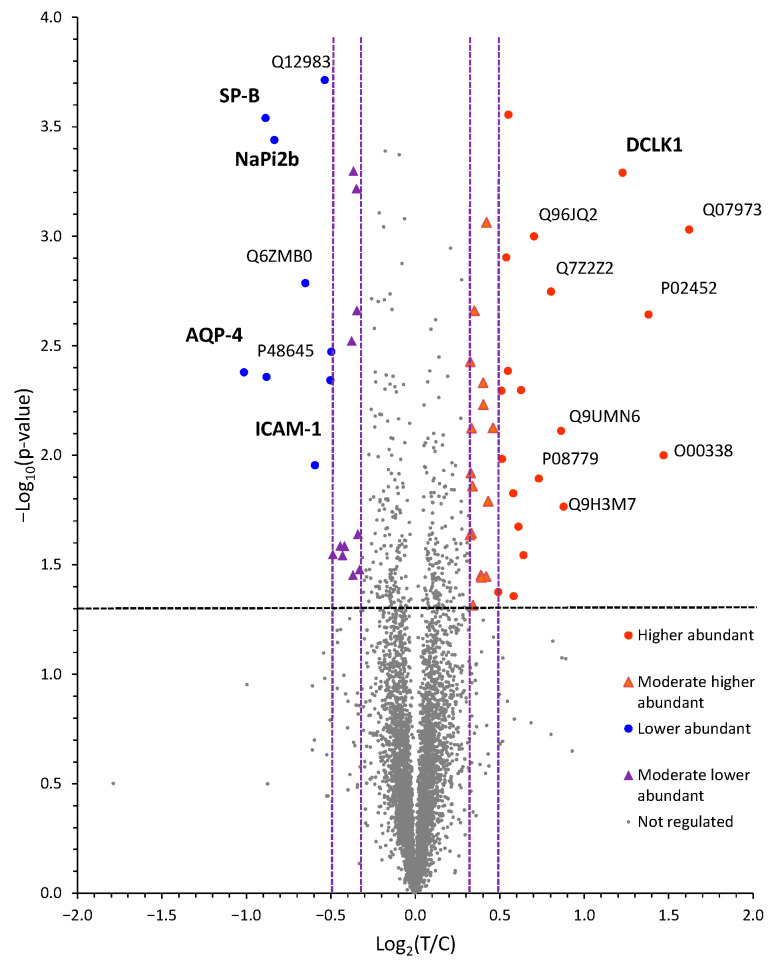
Protein abundance changes quantified by isobaric labeling and LC-MS/MS upon treatment of H44 cells with VD_3_. The volcano plot shows the relative changes of protein abundances: x-axis: log_2_ treatment (T) vs. control (C), the values correspond to the T/C values shown in Supplemental Tables S1–S4 y-axis: log_10_(*p*-value). Values above the horizontal dashed line are significant (α = 0.05). Protein abundance changes are classified into four groups: lower abundant; moderately lower abundant; moderately higher abundant; and higher abundant (indicated by vertical dashed lines from left to right). Differentially abundant proteins are annotated by colored labels, naming according to protein identifiers or abbreviations (Supplemental Tables S1–S4; for reasons of readability, only selected proteins are labeled).

Using this untargeted proteomics approach, we identified more than 7200 proteins (Supplemental Tables S1–S5) employing strict identification criteria, i.e., at least two tryptic peptides with at least one proteotypic peptide. Quantification of 6455 proteins was achieved using these quality criteria. Statistical treatment of the quantification data finally revealed 365 proteins as differentially abundant between treatment and control, of which 56 proteins were identified as significantly increased or decreased in abundance following VD_3_ (10 nM) treatment (Figure 7; Supplemental Tables S1–S4). We further classified the proteins according to the following filter criteria: (i) higher abundant (log_2_(T/C) ≥ 0.485; fold change: T/C > 1.4), (ii) moderately higher abundant (0.485 > log_2_(T/C) ≥ 0.321; fold change: 1.40 > T/C ≥ 1.25), (iii) lower abundant (log_2_(T/C) ≤ −0.485; fold change: T/C ≤ 0.71), and (iv) moderate lower abundant (−0.485 < log_2_(T/C) ≤ −0.321; fold change: 0.71 < T/C ≤ 0.80).

Of the 56 proteins, 20 showed higher abundances, 16 were moderately higher abundant, whereas 9 proteins were reduced in abundance, with 11 more showing moderate reduction in abundance.

These 56 proteins are associated with a broad range of cellular functions, including constitutional extracellular matrix components, cellular transport proteins, surfactant proteins, lipid metabolism, and immune-regulatory elements.

### 2.6. Verification of VD_3_-Regulated Target Molecules in NCI-H441 Identified by Proteomics

From proteins showing significant changes within the proteomics analysis, a number are known to be involved in cellular transport processes and immune defense of alveolar epithelial cells but are also molecules that may play a role in barrier formation. These were further analyzed and verified in terms of their VD_3_-dependent expression.

**Figure 8:**
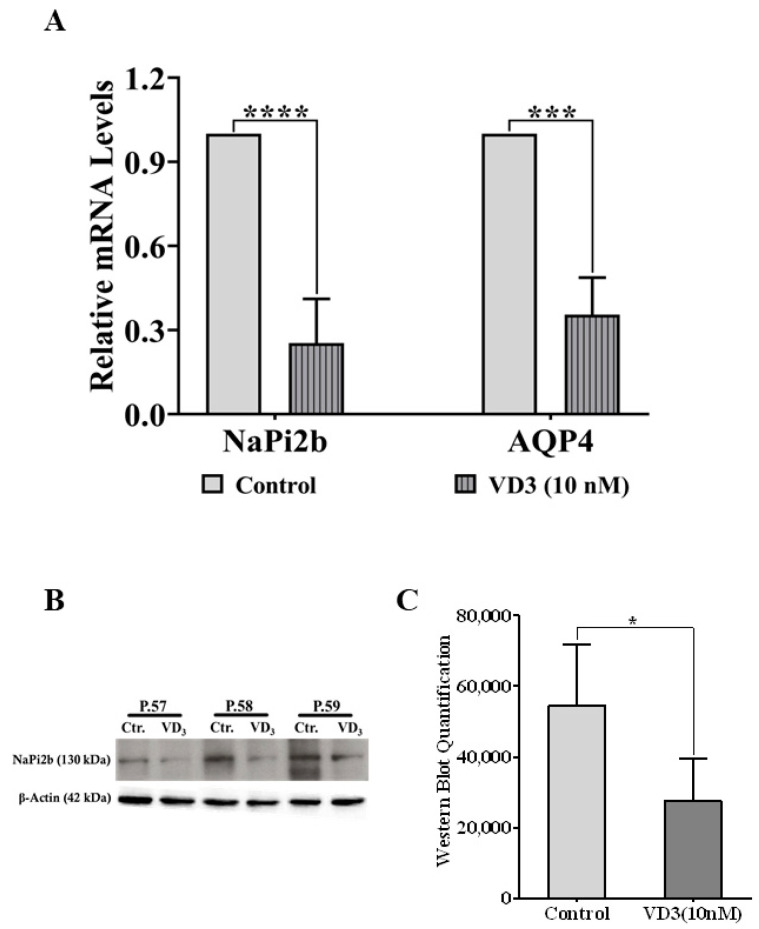
Expression of the cell membrane transport proteins NaPi2b and AQP4 in NCI-H441 cells. (**A**) mRNA levels of NaPi2b and AQP4 in NCI-H441 cells evaluated through real-time PCR from the mono-culture of NCI-H441 cells with dexamethasone (1 µM) and VD_3_ (10 nM) treatments (day 7). (**B**) Western blot of NaPi2b from the mono-culture of NCI-H441 cells with dexamethasone (1 µM) and VD_3_ (10 nM) treatment (day 7). (**C**) Quantification of the Western blots for NaPi2b in NCI-H441 cells. The values were shown as mean values ± SD from three independent experimental sets with the passage numbers of NCI-H441 cells between 57 and 59 (*n* = 3). * *p* ˂ 0.05, *** *p* ˂ 0.001, **** *p* ˂ 0.0001, one- and two-way ANOVA.

Amongst those, the gene expression levels of the transport proteins NaPi2b and AQP4 showed consistent results to the proteome data. In this context, VD_3_ significantly inhibited the mRNA levels of both molecules (Figure 8A), and the impact of VD_3_ (10 nM) on the sodium-dependent phosphate transporter NaPi2b was also verified by Western blotting (Figure 8B,C).

**Figure 9:**
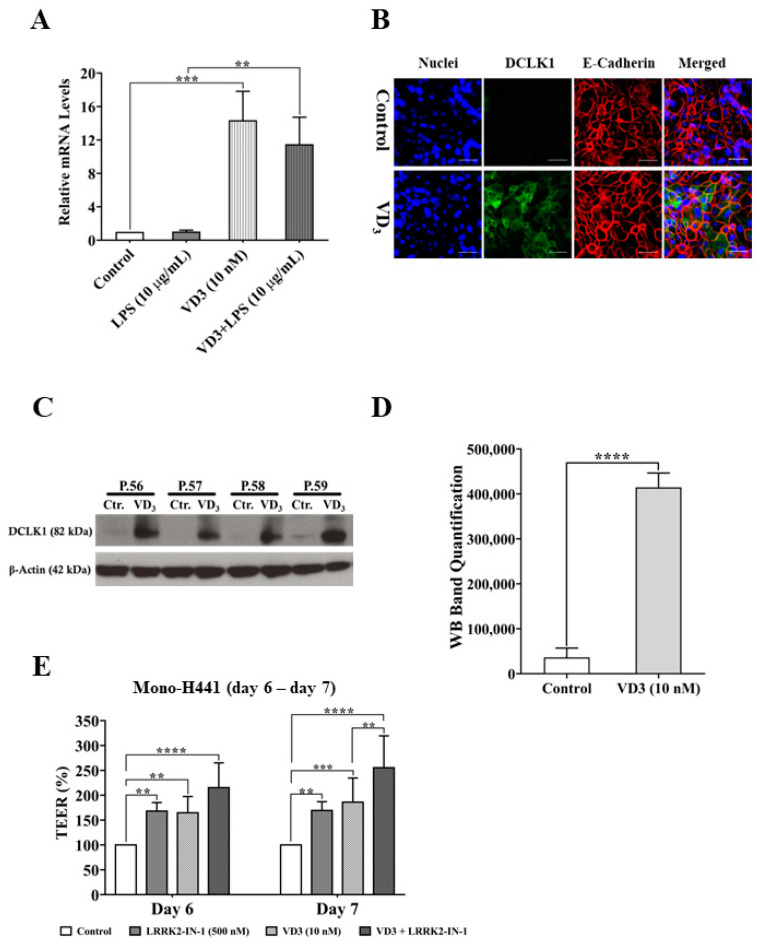
Expression of DCLK1 in the NCI-H441 cells. The expression of DCLK1 was analyzed from the mono-culture of NCI-H441 cells with dexamethasone (1 µM) and VD_3_ (10 nM) treatments and 7 days of cell culture. (**A**) mRNA levels of DCLK1 in NCI-H441 cells evaluated through real-time PCR. The cells were pretreated with VD_3_ (10 nM) and treated with LPS (10 µg/mL) on day 5 for 48 h. (**B**) Immunofluorescence analysis of DCLK1 in NCI-H441 cells after VD_3_ (10 nM) treatment and 7 days of culture. Blue: nuclei, green: DCLK1, red: E-cadherin, magnification: 40 folds, scale bar: 50 µm. (**C**) Western blot of DCLK1 from the mono-culture of NCI-H441 cells after VD_3_ (10 nM) treatment and 7 days of culture and (**D**) quantification. (**E**) Relative TEER values (%) of NCI-H441 cells treated with VD_3_ (10 nM) and DCLK1 inhibitor LRRK2-IN-1 (500 nM) on days 6 and 7. The values were shown as mean values ± SD from at least three independent experimental sets with the passage numbers of NCI-H441 cells between 56 and 59 (*n* ≥ 3). ** *p* ˂ 0.01, *** *p* ˂ 0.001, **** *p* ˂ 0.0001, one-way ANOVA, and *t*-test.

Another protein that was higher abundant after VD_3_ treatment in NCI-H441 cells is doublecortin-like kinase 1 (DCLK1). This protein belongs to the family of serine/threonine protein kinases and is associated with the regulation of microtubules in the cytoskeleton [32] but has also been reported to be associated with epithelial barrier function in the gut. To the best of our knowledge, a VD_3_-dependent alteration of DCLK1 abundance has not been reported before. The significantly increased amount of DCLK1 by VD_3_ in NCI-H441 cells was also verified on the gene (Figure 9A), protein (Figure 9C,D), and morphological levels (Figure 9B).

In order to determine a potential impact of DCLK1 on NCI-H441 barrier function, we performed initial experiments using the DCLK1 inhibitor LRRK2-IN-1 and assessed its influence on the barrier of NCI-H441. Notably, the inhibitor increased the barrier of epithelial cells, and the combination of LRRK2-IN-1 and VD_3_ showed the highest impact on epithelial barrier (Figure 9E), indicating the involvement of DCLK1 in the barrier regulation, although the detailed mechanisms need further evaluation.

## 3. Discussion

Cellular and molecular mechanisms leading to the dysfunction of the alveolar–capillary barrier in the lung in the case of infection and inflammation are multifactorial processes. Thus, therapeutical options to preserve or reconstitute the functionality of the alveolar–capillary barrier in the case of acute lung injury and ARDS still resemble a major clinical challenge.

The active form of vitamin D_3_, 1α, 25-dihydroxyvitamin D3 VD_3_, influences a variety of biological processes such as inflammation, infection, and barrier functionality. In this context, VD_3_ deficiency is associated with a higher risk for ALI/ARDS [12] as well as involved in many other lung diseases [14,33,34], and it causes a higher mortality in critically ill patients [35,36]. Nevertheless, due to the multifactorial action of VD3 and the complexity of VD_3_-triggered pathways, the cell type-specific effects of VD_3_ in the lung, the involved molecular mediators, and the associated mechanism are not fully understood. Beyond that, the identification of VD3-responsive target molecules involved in maintaining or restoring the alveolar–capillary barrier of the lung might lead to new concepts to treat ALI/ARDS as well as other diseases in the lung.

In our previous study, we have shown that the inflammatory cytokines secreted by microvascular endothelial cells are some of the major factors resulting in epithelial barrier breakdown when the alveolar–capillary barrier is exposed to LPS from the endothelial compartment [29,30]. In this present study, VD_3_ increased epithelial barrier formation in NCI-H441 in both mono- and co-cultures with OEC, as indicated by TEER measurement, and was able to protect the barrier in the case of exposure to IL-6 and different doses of LPS ranging from 100 ng/mL to 10 µg/mL. This protective effect of VD3 on the alveolar–capillary barrier is quite remarkable and covers a wide LPS concentration range in comparison to the previously reported protective effect of the surfactant component POPG (16:0/18:1-palmitoyloleoyl-phosphatidylglycerol (POPG)), which was restricted in this in vitro model to LPS concentrations of 100 ng/mL [30].

In contrast to the effect on the epithelial cell barrier, no effect of VD3 on OEC resembling the microvascular component was observed in impedance measurements. Therefore, these data suggest that the alveolar epithelial cells are the main target for the VD3-mediated alveolar–capillary barrier protection, although reports in the literature also have shown an impact of VD3 on the angiopoietin tie-2 pathway associated with vascular permeability in the lung [37]. Furthermore, VD3 seems to induce distinct mechanisms in epithelial cells leading to the regulation of their barrier properties [38,39]. Although both cell types, endothelial and epithelial cells, regulate their paracellular barrier properties via tight junctional and adhesion junctional complexes, different constitutional molecules in the junctional complexes and different regulatory mechanisms for VD3 in the cell types exist. Although an impact of VD3 on the expression rate of tight junctional molecules in lung tissue lysates has been reported before by other groups [19], our data did not show any indication that VD3 increases the expression of tight junctional molecules such as ZO-1 (zonula occludens protein 1) or occludin-1. In addition, adaptation of biological barriers in response to a series of stimuli is supposed to be regulated via phosphorylation and dephosphorylation of junctional molecules to allow the fast adaptation process.

In addition, we addressed in this study whether VD3 can modulate the inflammatory response and the secretion of inflammatory cytokines in the epithelial or endothelial cells. As shown here and in previous studies, inflammatory cytokines secreted by the endothelial cells in response to LPS impaired the barrier [29,30], so VD3-induced changes of inflammatory cytokines might have an indirect effect on the epithelial alveolar–capillary barrier. IL-6 is a key mediator in inflammation, and it also plays a significant role in the barrier property modulation of epithelial cells [40,41], as has been demonstrated also in this present study. Nevertheless, the positive impact of VD3 on the barrier in our study cannot be explained by a reduced expression of inflammatory cytokines such as IL-6 or IL-8 mainly derived from endothelial cells, as indicated in the mono-culture and co-culture set ups. On the other hand, we observed decreased levels of IL-6 in the apical/epithelial compartment in VD3-pretreated co-cultures. These data suggest a restricted diffusion of IL-6 from the endothelial to the epithelial compartment as a consequence of increased epithelial barrier properties in response to VD_3_.

Further, inflammatory cytokines released by endothelial cells triggered surfactant protein A (SP-A) expression in NCI-H441 cells, whereas the direct exposure to the LPS shows no effect. SP-A is classified as a defense molecule in the innate immune system of alveolar epithelial cells [40,42,43] and facilitates the defense of lung epithelial cells against LPS and attenuates the inflammation-induced lung injury [40,41]. Our results displayed a significant suppression of the SP-A gene expression in NCI-H441 cells by VD_3_, which refers to the regulation through vitamin D/VDR signaling pathways. However, the expression of SP-A in NCI-H441 cells is reported to be dose dependent, which can be induced by higher doses of VD_3_ from 20 nM [44].

Another defense molecule triggered by VD3 in our study is the antimicrobial peptide cathelicidin LL-37. LL-37 can neutralize LPS [45] and induces multiple immunomodulatory mechanisms during the defense against pathogen invasions and infections [46,47]. LL-37 is well known to be induced by VD3 in immune and epithelial cells, but it also modulates wound healing [48] and might thus have a direct effect on barrier and epithelial cell function.

Based on the data in this study confirming the induction of LL-37 by VD3 in NCI-H441 cells, we tested the direct effect of LL-37 on the properties of the epithelial barrier. The experimental set up was also in accordance with the low concentrations as detected by ELISA (0.1 ng/mL, 1 ng/mL, 10 ng/mL). The results revealed a distinct effect of LL-37 on the barrier of epithelial cells in a dose-dependent manner (Figure 6B). Furthermore, LL-37 (10 ng/mL) could significantly maintain the function of the alveolar–capillary barrier in the co-culture model in response to the LPS. In this context, LL-37 has been demonstrated to increase the stiffness of lung epithelial cells, including A549 and primary lung epithelial cells, and enrich the abundance of the cytoskeleton component, F-actin, to prevent the bacterial invasion [49]. Therefore, it can be concluded that the VD3-mediated induction of LL-37 is at least partly responsible for maintaining the epithelial barrier in response to LPS, which is in accordance with reports for gut epithelial cells [50].

Proteome analysis in this study identified several target molecules regulated by VD3 in epithelial cells, such as the sodium-dependent phosphate transporter NaPi2b, water channel protein aquaporin 4 (AQP4), and doublecortin-like kinase 1 (DCLK1), amongst which DCLK1 has not yet been reported to be VD3-dependent.

The study indicated lower abundances of NaPi2b and AQP4 and higher abundance of DCLK1 in NCI-H441 cells treated by VD_3_ (10 nM) through the proteomic approach (MS/MS), which were also verified on the gene expression and protein levels.

Although NAPi2b and AQP4 have been shown as the promoting factors for tumor progression, invasion, and metastasis [51,52,53,54], these two transporters mediate the homeostasis of cellular phosphate uptake [46,55] and water flow [45,47]. AQP4 is localized in the basolateral membrane of the lung airspace epithelium [56] and is involved in the edema resolution after lung injury [57]. Although these two molecules mediate important processes in lung fluid regulation and surfactant metabolism, no direct impact on the tight junctional barrier functionality in epithelial cells has been reported for AQP4 and NAPi2b.

In contrast to this, DCLK1, a calmodulin-dependent protein kinase, is associated with Ca^2+^ signaling pathways and the regulation of microtubule polymerization in the cytoskeleton [32,49]. Although in a DCLK1 knockout mouse model the positive effect of DCLK1 on the tissue barrier preservation in the gut is reported [58], the concrete mechanisms regarding the functions and effects of DCLK1 on the lung airspace barrier or other barriers are still elusive.

DCLK1 inhibition by LRRK2-IN-1 in the presence of VD3 resulted, even in our initial studies, in an increase of epithelial barrier function, indicating a complex role of DCLK1 in epithelial barrier regulation, which still needs to be further clarified. Beyond this direct role for barrier function, DCLK1 expression has been reported as a marker to indicate specific epithelial cell types, tuft cells, found in epithelial tissues such as the gut [59]. In the lung, these cell types have been referred to as brush cells [60,61], or in rat lung tissue as type 3 pneumocytes, characterized by villin and CK18 [62]. In the lung, brush cells are chemoreceptors regulating breathing or suggested to be involved in regulating capillary resistance and perfusion during hypoxia [63,64,65].

A common feature of tuft in the gut or brush cells (BC) in the lung is that they are supposed to support stem cell function or epithelial maintenance and repair. DCLK1-positive tuft cells seem to derive from lrp5-positive epithelial stem cells in the gut [66]. These findings, along with the recent report that lgr5 intestinal stem cell function is dependent on vitamin D3 [67], are in line with this present study indicating a correlation of vitamin D3 and DCLK1 in lung epithelial cells. In this present study, this correlation was evidenced on several levels of investigation and using several methods.

In the lung, brush cells are increased in the context of pneumothorax and respiratory distress [68]. Furthermore, bleomycin-induced pneumonitis also resulted in an increase of BC, probably associated with repair of the epithelium after bleomycin-induced injury [64,65]. DCLK1-positive tuft cells in the gut also seem to increase in the context of infections and were reported to fulfill key functions in inflammation [69] by interacting with cells from the immune system.

Considering these reports from the literature, the functional role of vitamin D3 and DCLK1 in epithelial barrier function and repair has to be further defined in the individual cell types of the lung epithelium.

## 4. Materials and Methods

### 4.1. Cells and General Cell Culture

#### Reagents 

1α, 25-dihydroxyvitamin D3 (VD3) (Sigma-Aldrich, St. Louis, MO, USA) was reconstituted with 95% ethanol, and LRRK2-IN-1 (Calbiochem, Darmstadt, Germany) was reconstituted with dimethyl sulfoxide (DMSO). Other compounds and peptides used in the study including lipopolysaccharide (LPS) (Sigma-Aldrich, MO, USA), interleukin-6 (IL-6) (PeproTech, Cranbury, NJ, USA), and LL-37 (Tocris Bioscience, Bristol, UK) were purchased as indicated.

The human bronchial adenocarcinoma epithelial cell line, NCI-H441 cells, was used as the model of ATII cells. In addition, another lung adenocarcinoma epithelial cell line, A549 type 2 cells, was used as additional reference in parts of the experiments. The cells were cultured and maintained in RPMI 1640 medium (Gibco^®^/Life Technologies, Paisley, UK) supplemented with 10% fetal bovine serum (FBS) (Sigma-Aldrich, Lenexa, KS, USA), 1% penicillin/streptomycin (Biochrom GmbH, Berlin, Germany), and 1% L-Glutamine (Gibco^®^/Life Technologies, Paisley, UK) at 37 °C with 5% CO_2_. The passage numbers of NCI-H441 cells ranged from 55 to 60.

Outgrowth endothelial cells (OECs) displaying the character of microvascular endothelial cells were isolated from the mononuclear cell (MNC) fraction of human peripheral blood with the approval of local ethics committees and the consent of the individual donors according to the standard protocol established by Fuchs et al. [70,71,72]. OECs were cultured and maintained in endothelial growth medium-2 (EGM-2) (Lonza, Walkersville, MD, USA) supplemented with the growth factor kit, 7% FBS, and 1% penicillin/streptomycin at 37 °C with 5% CO_2_. The passage numbers of OECs used for this study ranged from 5 to 16.

### 4.2. Mono- and Co-Culture of NCI-H441 Cells with OECs for Barrier Property Assessment

For the determination of barrier properties, Transwell^®^ inserts (Corning Costar, Cambridge, MA, USA) were used for the mono-cultures of NCI-H441 cells and co-cultures of NCI-H441/OECs manufactured with semi-permeable polyester filters with micropores (filter area: 0.33 cm^2^, pore size: 0.4 μm). In the mono-culture, NCI-H441 cells were seeded in the 200 μL RPMI 1640 on the apical side of the filters (1.5 × 10^5^ cells/cm^2^). In the co-culture of NCI-H441 with OECs, the basolateral side of the Transwells^®^ coated with fibronectin (10 μg/mL) (Millipore, Bedford, CA, USA) were first seeded with OECs (5 × 10^4^ cells/cm^2^) and filled up with 800 µL EGM-2. Then, NCI-H441 cells were apically seeded as described above. A total of 3 days after the cell seeding, the cells were apically treated with dexamethasone (1 μM) (Sigma-Aldrich, St. Louis, MO, USA). The cells were pretreated with VD3 (10 nM) or LL-37 (10 ng/mL) both apically and basolaterally in the Transwells^®^ on day 3. The LPS (100 ng/mL, 1 µg/mL, 10 µg/mL) or IL-6 (10 ng/mL) and LRRK2-IN-1 (500 nM) treatments were implemented from day 5 to day 7 for 48 h. LPS and IL-6 were only applied basolaterally in the Transwells^®^ in both mono- and co-culture systems due to previous observations that only basolateral exposure and endothelium-derived factors resulted in effects on the epithelial barrier [29,30]. The cells were cultured at 37 °C and 5% CO_2_ with daily medium exchange from day 3 to day 7. At least three cell culture sets (*n* ≥ 3) with different passage numbers of the NCI-H441 cells and OECs from different donors were carried out.

### 4.3. Trans-Epithelial Electrical Resistance (TEER) Measurement

The TEER measurement was performed from day 3 to day 7 in the mono-cultures of NCI-H441 cells and co-cultures of NCI-H441/OECs in the Transwell^®^ inserts to determine the barrier integrity and bioelectrical resistance (Ω × cm^2^) of epithelial cells. The epithelial voltohmmeter, EVOM (World Precision Instruments, Sarasota, FL, USA), was applied for the TEER measurement equipped with a pair of STX-2 chopstick electrodes. Blank Transwell^®^ values were subtracted from measured values. Endothelial barrier properties that cannot be detected by EVOM result in very low values close to the background so that values from co-culture resemble epithelial barrier characteristics.

### 4.4. Endothelial Cell Barrier Monitoring

The barrier function of endothelial cells was determined using an ECIS (Electric Cell-Substrate Impedance Sensing) instrument. OECs were seeded on each well of the 8-well 10 gold film electrode array slide (5 × 10^4^ cells/cm^2^) (8W10E) (Applied Biophysics Inc., Troy, NY, USA) coated with fibronectin (10 μg/mL). The gold pads at the edge of array slides were connected to the array station of the ECIS instrument, which was placed in the incubator at 37 °C with 5% CO_2_. The real-time impedance values (Ω) of the cell barrier were monitored. The blank values of each well were subtracted from the measured values at different time points.

### 4.5. Immunofluorescence Staining and Imaging

To analyze the morphological effect of VD_3_ on the properties of the alveolar–epithelial barrier, immunofluorescence staining of tight junctions (TJs) and the associated molecule, zonula occludens-1 (ZO-1), in NCI-H441 was performed. NCI-H441 cells cultured on the 8-well ibidi slides (ibidi GmbH, Martinsried, Germany) were pretreated with VD_3_ (10 nM) and dexamethasone (1 μM) on day 3. Then, the cells were cultured with the conditioned medium collected from the VD_3_- (10 nM) and LPS (100 ng/mL)-treated OECs from day 5 to day 7 for 48 h. On day 7, the cells were washed with PBS (Gibco, Darmstadt, Germany) and fixed with 4% paraformaldehyde (PFA) (Affymetrix, Santa Clara, CA, USA). The cells were then permeabilized with 0.5% Triton^®^X-100 (Sigma-Aldrich, Taufkirchen, Germany) and incubated with primary anti-ZO-1 antibody (1:100, rabbit) (Invitrogen, Carlsbad, CA, USA) for 1.5 h at room temperature. Afterwards, the cells were incubated with secondary Alexa Fluor 555 anti-rabbit antibody (1:1000, donkey) (Invitrogen Molecular Probe, Eugene, OR, USA) for 1 h. The cell nuclei were counterstained with Hoechst (Invitrogen, Eugene, OR, USA). Samples were imaged using a Zeiss LSM 510 Meta confocal laser scanning microscope (Carl Zeiss Advanced Imaging Microscopy, Jena, Germany). For the DCLK1 staining, the cells were solely treated with VD_3_ (10 nM) and dexamethasone (1 μM) from day 3 to day 7. The primary antibodies of DCLK1 (DCAMKL1 1:5000, rabbit) (Abcam, Cambridge, UK) and E-cadherin (1:40, goat) and secondary antibodies Alexa Fluor 488 anti-rabbit (1:1000, donkey) and Alexa 555 Fluor anti-goat (1:1000, donkey) (Invitrogen, Eugene, OR, USA) were applied.

### 4.6. Semi-Quantitative Real-Time Polymerase Chain Reaction (Real-Time PCR)

On day 7 of the cell culture, the NCI-H441 cells from the apical side of the Transwells^®^ and 24-well plates and OECs from 24-well plates were harvested for the RNA isolation using the peqGOLD Total RNA kit according to the manufacturer’s protocol (Peqlab, Darmstadt, Germany). Then, 1 μg of the extracted RNA was transcribed into cDNA using the High-Capacity RNA-to-cDNA Kit according to the standard protocol from the manufacturer (Applied Biosystems, Carlsbad, CA, USA). The relative gene expression was quantified by semi-quantitative real-time PCR using the Rotor-Gene Q (Qiagen, Hilden, Germany) detection system. For one reaction, 12.5 μL QuantiTect^TM^ SYBR^®^ Green PCR Master Mix (Qiagen, Hilden, Germany) and 2.5 μL QuantiTect^TM^ SYBR^®^ Green Primer Assay (RPL13A, IL-1β, IL-8, SP-A, SP-D; all provided by Qiagen, Hilden, Germany) or 1 μL of 10 μM forward + reverse primer (IL-6, provided by Eurofin; LL-37 provided by Sigma/GENOSYS), 4 μL cDNA (10 ng/μL), and Nuclease-Free water (Ambion^®^ Thermo Fisher Scientific, Pleasanton, CA, USA) was used to adjust to the final volume to 25 μL. The real-time PCR was performed with the following cycler program: 95 °C for 2 min and 40 cycles of denaturation at 95 °C for 15 s annealing and elongation at 60 °C for 1 min. A housekeeper gene of ribosomal protein 13A (RPL13A) was taken as an endogenous standard to normalize the data using the ΔΔCt method during the gene expression assessment. The value of control samples was set as 1.

### 4.7. Enzyme-Linked Immunosorbent Assay (ELISA)

The medium supernatants from both apical and basolateral sides of the co-cultures in the Transwell^®^ inserts and mono-cultures in the 24-well plates were collected on day 7 for the ELISA. The concentrations of IL-6 and IL-8 in the supernatants were analyzed using the Human IL-6 ELISA kit and Human IL-8 ELISA kit, respectively, according to the manufacturer’s protocols (R&D Systems, Minneapolis, MN, USA).

To determine the secretion levels of LL-37 from NCI-H441 cells, the modified mono-culture of NCI-H441 cells on the 24-well plates was applied using extended cell culture cycle to 15 days and medium exchange frequency of every 4 days. The medium supernatants were collected on days 7, 11, and 15. The LL-37 concentrations were assessed using the Human LL-37 ELISA kit according to the instructions from the manufacturer (Hycult Biotech, Uden, The Netherlands). The optical density of each well was detected using an automatic microplate reader (Apollo LB 911 TECAN, Berthold Technologies, Bad Wildbad, Germany).

### 4.8. Sample Preparation for Quantitative Proteome Analysis

H441 cells were seeded at a concentration of 0.15 × 10^6^ cells/cm^2^ in culture medium (RPMI 1640 containing 10% FBS, 100 U/mL penicillin, 100 µg/mL streptomycin and 20 µM of L-glutamine) in T75 cell culture flasks. Three biological replicates using different cell passages for both control and VD3-treated samples were cultivated. Three days post seeding, cells were treated with VD3 and dexamethasone as described above and harvested on day 7 for proteome analysis. After medium removal, cells were washed twice with 5 mL PBS and incubated for 5 min at 37 °C with 5 mL trypsin/EDTA (0.05%; Sigma-Aldrich, Taufkirchen, Germany). After detachment, the cell pellet was resuspended in 10 mL PBS and washed/centrifuged twice. Cells were lysed by resuspending in lysis buffer (50 mM TEAB, 1% SDS, one *cOmplete* protease inhibitor tablet (Roche, Penzberg, Germany) in 10 mL Milli-Q-water) on ice. For further lysis, cells were treated by sonication (Bandelin Sonoplus HD2070, Bandelin electronic, Berlin, Germany) for 10 s (65% power). Lysates were transferred to 1.5 mL Eppendorf tubes and centrifuged at 21,000× *g* at 2 °C for two hours. The supernatant (soluble fraction) was used for further proteome analysis. Protein concentration was determined by standard BCA assay (Pierce BCA Protein Assay Kit, Thermo Scientific, Darmstadt, Germany).

Aliquots containing 120 µg protein per biological replicate were precipitated using methanol/chloroform [73] with minor alterations (organic solutions were at −20 °C, and the precipitation was performed at 4 °C). The protein pellets were redissolved in 20 µL 100 mM triethylammonium-bicarbonate buffer (TEAB)/0.25% RapiGest (Waters, Saint-Quentin, France).

Proteins were reduced by addition of 5 µL of 50 mM Tris-(2-carboxyethyl)-phosphin (TCEP) in 100 mM TEAB buffer (final TCEP concentration: 10 mM) and incubation at 55 °C for one hour. Cysteine groups were protected by carbamidomethylation at room temperature by addition of 5 µL of 300 mM 2-chloroacetamide (CAA) solution in 100 mM TEAB buffer (final CAA concentration 50 mM) and incubation for 30 min in the dark. Residual CAA was deactivated by exposure to light for 30 min.

Proteins were proteolytically processed using trypsin (sequencing grade; modified, Promega, Madison, WI, USA) at a protease-to-total protein ratio of 1:50 (*w*:*w*) in 100 mM TEAB overnight at 37 °C. Peptides were derivatized using the tandem mass tags (TMT)-sixplex reagents set (Thermo Scientific, Darmstadt, Germany) according to the manufacturer’s protocol; labeling scheme: control biological replicate 1-3: TMT-126, 128, 130; treatment replicate 1-3: TMT-127, 129, 131. After combining the six different reaction channels (control/treatment, three biol. replicates each), 5 µL 95% formic acid was added for hydrolysis of RapiGest (1 h, RT), followed by centrifugation (14,000× *g*, 20 min, 4 °C). The supernatants were collected and freeze dried.

TMT-labeled peptides were separated and analyzed by a two-dimensional high pH/low pH reversed phase separation scheme [74] with the second dimension coupled online to electrospray mass spectrometry.

First dimension separation was performed employing a Gemini C18 column (3 µm, 110 Å, 150 × 2 mm, Phenomenex, Aschaffenburg, Germany) on an Ultimate 3000 HPLC (Dionex, Dreieich, Germany). Eluent A: 72 mM triethylamine, 0.35% *v*/*v* acetic acid in Milli-Q-water, pH 10; eluent B: 72 mM triethylamine, 0.35% *v*/*v* acetic acid in acetonitrile. Gradient: 2 min isocratic at 2% eluent B, linear gradient to 5% up to 5 min, linear gradient to 55% up to 55 min, then linear to 90% eluent B up to 60 min; flow rate: 200 µL/min. One-minute fractions were collected from minute three until the end of the separation. Inter-run washing/equilibration: 9 min isocratic at 90% eluent B; 1 min linear to 5%, 10 min isocratic at 2%.

Fractions were concatenated [75], leading to 7 pooled fractions according to the following scheme: pool 1: fraction 1, 8, 15, 22, 29, 36, 43; pool 2: fraction 2, 9, 16, 23, 30, 37, 44, 50; accordingly, up to pool 7: fraction 7, 14, 21, 28, 35, 42, 49, 56. Pools were dried by freeze drying and redissolved in loading buffer (3% acetonitrile, 0.1% trifluoroacetic acid in Milli-Q-water) prior to injection.

Second dimension separation of pools 1–7 was performed using ion-pairing reversed-phase chromatography employing a UHPLC (Ultimate 3000 RSLC nano System, Dionex, Sunnyvale, CA, USA) coupled online to a mass spectrometer (see below). Samples were loaded via an autosampler using 1 µL per injection. Precolumn: Acclaim PepMap100 C18 (5 μm, 100 Å, 300 μm × 5 mm). Column: Acclaim PepMap RSLC analytical column (2 μm, 110 Å, 75 μm × 500 mm, nanoViper; Thermo Fisher Scientific, Dreieich, Germany); flow rate: 300 µL/min; eluent A: 0.05% formic acid in Milli-Q-water; eluent B: 0.05% formic acid, 80% acetonitrile in Milli-Q-water. Gradient: 2 min isocratic 4% eluent B; linear increase within 180 min to 40% eluent B; increase within 8 min to 90%, 10 min isocratic at 90%; linear decrease to 4% within 1 min; equilibration for 15 min.

Online MS analysis was performed on a Q Exactive Plus mass spectrometer (Thermo Fisher Scientific, Bremen, Germany). Measurements were performed in positive ion mode; MS/MS measurements were performed by higher energy collisional dissociation (HCD) in data-dependent mode. Parameters MS^1^: resolution: 70,000, scan range: 350–1400 *m*/*z*, maximum injection time: 50 ms, AGC target: 3 × 10^6^, spray voltage: 1.5 kV, capillary temperature: 250 °C. Parameters MS^2^: resolution: 17,500; scan range: 100–2000 *m*/*z*, isolation window: 1.2 *m*/*z*, maximum injection time: 100 ms, AGC target: 1 × 10^5^, Top 15, dynamic exclusion: 30 s, charge exclusion: unassigned, 1, ≥7; minimal intensity: 1 × 10^4^, use lock masses: best, exclude isotopes: on.

The seven pooled fractions from first dimension separation were each analyzed four times (technical replicate injections) in second dimension LC-MS. For the acquisition of the fourth technical replicate, an exclusion list (see below) was generated. Data were recorded in raw format and have been deposited to the ProteomeXchange Consortium [76] via the PRIDE partner repository with the dataset identifier PXD040622.

The first three replicate LC-MS runs of each pool were true technical replicates. The first two of these were processed and, after database search (see below), used to compile an exclusion list of *m*/*z* values representing the 5000 (software limited) high confidence peptides (FDR ≤ 0.01) with the highest Xcorr values. Corresponding *m*/*z* values were excluded from data-dependent MS/MS acquisition over a retention time window of 5 min and mass tolerance of 7 ppm for the fourth replicate of each sample to reduce redundant scan events and increase protein coverage.

Database search and protein quantification of the acquired RAW files were performed using Proteome Discoverer Software (Vers. 1.4.1.14; Thermo Fisher Scientific, Bremen, Germany). A human proteome dataset from UniProt (version 4 July 2016, canonical and reviewed sequences only, no isoforms) was supplemented with common contaminants (cRAP laboratory and contact dust contaminants, 4 July 2016) and used to search spectra with the search algorithms Mascot (v2.2; Matrix Science) and SequestHT. Parameters used were peptide length min. 6, max. 144 amino acids; max. missed cleavages: 2; protease specificity: semi-tryptic; precursor mass tolerance: 7 ppm; fragment mass tolerance: 0.04 Da. Dynamic modifications: Met-oxidation, TMT-sixplex: Tyr-TMT, methylation and acetylation of Lys and phosphorylation of Ser, Tyr and Thr; static modifications: Cys: carbamidomethylation, Lys: TMT-sixplex, N-Term.: TMT-sixplex.

Percolator (v1.2) was used for posterior error calculation combining the results of the database searches with a false discovery rate restricted to α = 0.01 by q-value. Single peptide identifications were excluded, and protein groups were assigned using strict parsimony rules. Only unique peptides were used for quantification and the peptide-TMT ratios were normalized by the median of protein ratios.

To minimize ratio compression and underestimation, reporter ion signals of PSM were corrected by relative precursor interference, as described previously [77].

Briefly, the sum of reporter ion intensities for each PSM was normalized to one. Isolation interference for each channel was assumed to be reflected by the median of the corresponding reporter ion intensity within the dataset. The normalized median intensities for each channel were multiplied by the percentage of spectra interference to calculate the corresponding interference of each TMT channel for each PSM. The result was subtracted from normalized reporter ion intensities of the corresponding TMT channel in each PSM. Due to this recalculation of normalized reporter ion intensities, negative values occurred at a frequency below 0.1%, and these were replaced by the minimum positive value in each channel.

Perseus software (Version 1.5.5.3, [78]) was used for statistical analysis of the dataset (www.maxquant.org). Normalized data of each TMT channel were tested by Welch *t*-test with permutation-based FDR (1.000 cycles, α = 0.05, s_0_ = 0). Further, volcano plots (log_2_(treatment/control) vs. −log1_0_(*p*-value)) were generated.

Protein groups were imported in Perseus and Gene ontology (GO) groups, molecular function (GOMF), cellular compartment (GOCC), and biological process (GOBP) [79]. KEGG annotations (Kyoto Encyclopedia of Genes and Genomes, [80]) and protein families (pfam) [81]) were assigned. One-dimensional enrichment analysis was performed based on relative protein abundances (log_2_(treatment/control) (FDR, α = 0.05) [82].

### 4.9. Western Blot

To obtain protein for Western blot analysis, NCI-H441 cells were cultured in the T75 flasks with VD_3_ (10 nM) treatment. The cell culture was conducted as described before. On day 7, the cells were lysed with RIPA buffer (150 mM NaCl, 1% NP-40, 0.5% sodium deoxycholate, 0.1% SDS, 50 mM Tris, pH 8.0) supplemented with Protease Inhibitor Cocktail (Thermo Fisher Scientific, IL, USA) and Phosphatase Inhibitor Cocktail (Cell Signaling Technology, Danvers, MA, USA). The protein extracts were collected after centrifugation at 14,000× *g* and 4 °C, and concentrations were determined via the Pierce^TM^ BCA protein assay kit (Thermo Fisher Scientific, Rockford, IL, USA). Equal amount of the protein extracts was denatured and loaded using LDS Sample Non-Reducing Buffer (Thermo Fisher Scientific, Rockford, IL, USA) on the Novex^TM^ WedgeWell^TM^ 4–20% Tris-Glycine Gels (Invitrogen, Calsbad, CA, USA) to separate the proteins. Then, proteins were transferred onto the PVDF membranes (Millipore, Bedford, MA, USA) blocked with 3% skimmed milk (BIO-RAD, Hercules, CA, USA). The primary antibodies of NaPi2b (1:1000, rabbit) (Cell Signaling Technology, Danvers, MA, USA), DCLK1 (DCAMKL1, 1:5000, rabbit) (Abcam, Cambridge, UK), and β-actin (1:1000, rabbit) (Cell Signaling Technology, Danvers, MA, USA) were applied. The secondary antibody anti-rabbit IgG-HRP (1:5000, goat) (Santa Cruz Biotechnology, Dallas, TX, USA) was used for the detection. The membranes were washed with TBS/T buffer and exposed to Pierce ECL Western Blotting Substrate (Thermo, Rockford, IL, USA). The signals were detected using High Performance Chemi-Luminescence Films (GE healthcare, Little Chalfont, UK). Image Studio Lite (LI-COR, Lincoln, OR, USA) was used for quantification of the protein bands.

### 4.10. Statistical Analysis

The data from the experiments were analyzed using GraphPad Prism 7.0 (GraphPad Software, La Jolla, CA, USA). The data in all of the graphs were presented as mean ± standard deviation of the mean (SD). The data statistical analyses were performed through *t*-test and one- or two-way ANOVA. The statistical confidence level was set to be significant when the *p*-values were less than 0.05 (* *p* < 0.05, ** *p* < 0.01, *** *p* < 0.001, **** *p* < 0.0001). Please also refer to the individual descriptions for the different assays.

## 5. Conclusions

In this study, VD_3_ strengthened the barrier function of alveolar epithelial cells and protected the alveolar–capillary barrier from LPS- and IL-6-mediated dysfunction mainly by a direct impact on the epithelial cells. The barrier strengthening of epithelial cells was supported by the VD_3_-induced antimicrobial peptide LL-37 leading to higher TEER values in NCI-H441 and protecting the alveolar–capillary barrier when exposed to LPS. Finally, MS-MS-based proteomics identified VD3-dependent expression rates in alveolar epithelial cells, including a new target of VD3, the doublecortin-like kinase DCLK1. The influence of this molecule on epithelial barrier functionality was confirmed in the first experiments.

## Data Availability

Proteomics data are deposited at Proteome X Change with the accession PXD040622.

## Supplementary Materials

The supporting information can be downloaded at: https://www.mdpi.com/article/10.3390/ijms24087298/s1.
