# Supplementary material for: Alveolar–Capillary Barrier Protection In Vitro: Lung Cell Type-Specific Effects and Molecular Mechanisms Induced by 1α, 25-Dihydroxyvitamin D3
Source: Int J Mol Sci. 2023 Apr 14;24(8):7298. doi: 10.3390/ijms24087298 (PMC10138495; doi:10.3390/ijms24087298)
Supplement: Supplementary file 1 [file ijms-24-07298-s001.zip › ijms-2319618-supplementary/Supplemental_proofs/ijms-2319618-supplementary Figures_SF.docx]

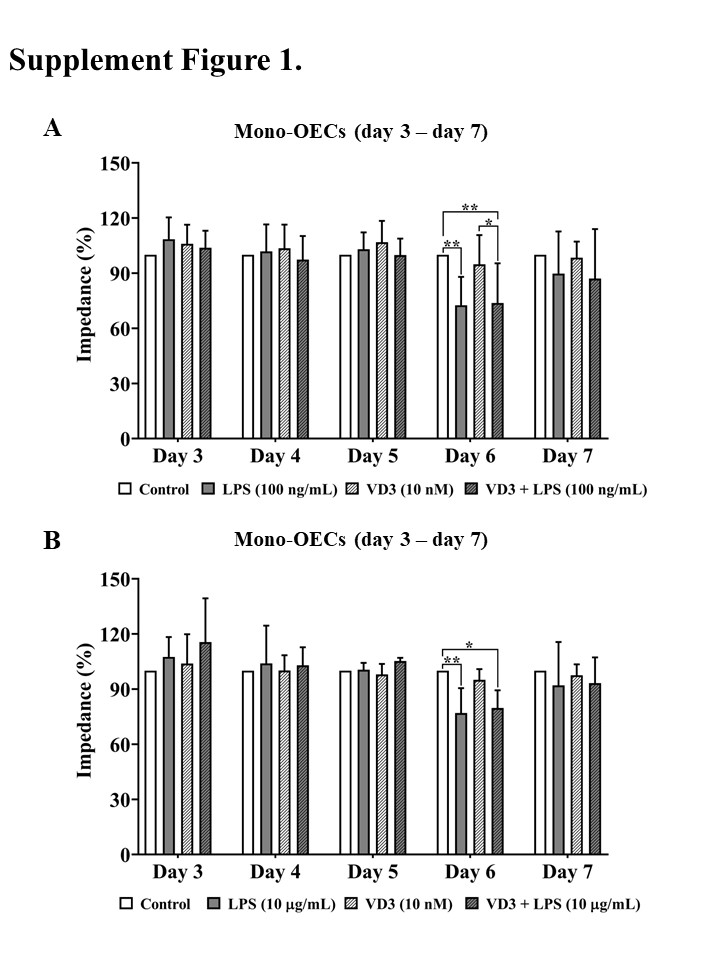


**Figure S1**.: Barrier properties of OEC treated with 100 ng/ml LPS **(A)** or 10µg/ml LPS **(B)** and VD3, * *p* ˂ 0.05, ** *p* ˂ 0.01, , two-way ANOVA.


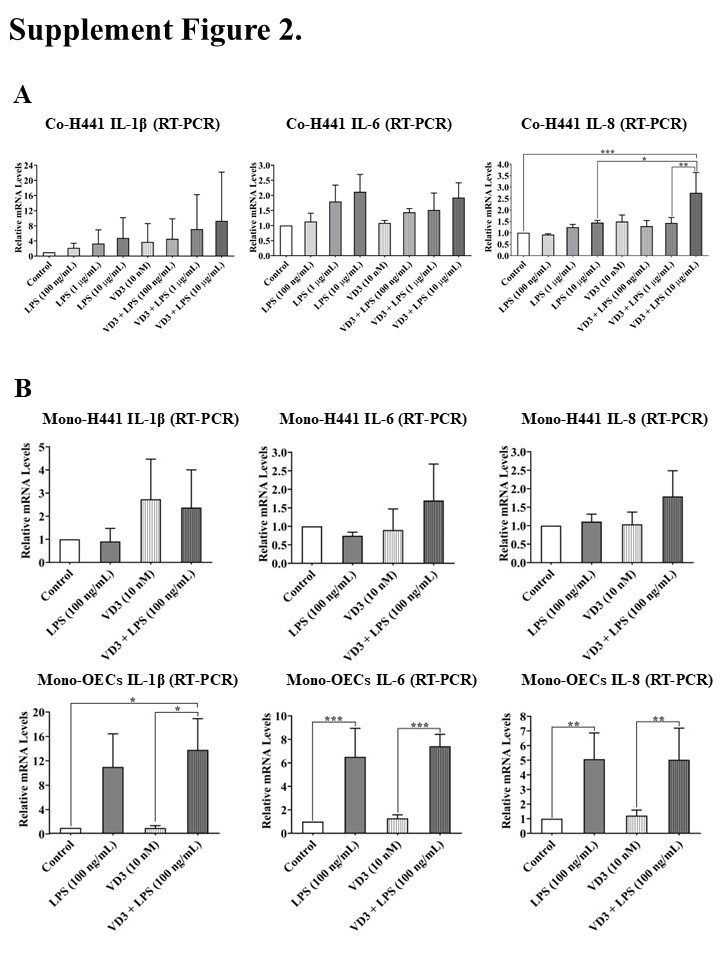


**Figure S2.**: Gene expression analyses of inflammatory cytokines IL-1ß and IL-6 in **(A)** H441 cells in co-culture with OEC or in **(B)** monocultures of H441 or OEC, * *p* ˂ 0.05, ** *p* ˂ 0.01, *** *p* ˂ 0.001, one-way ANOVA.
