# Supplementary material for: Alveolar–Capillary Barrier Protection In Vitro: Lung Cell Type-Specific Effects and Molecular Mechanisms Induced by 1α, 25-Dihydroxyvitamin D3
Source: Int J Mol Sci. 2023 Apr 14;24(8):7298. doi: 10.3390/ijms24087298 (PMC10138495; doi:10.3390/ijms24087298)
Supplement: Supplementary file 1 [file ijms-24-07298-s001.zip › ijms-2319618-supplementary/Supplemental_proofs/Suppl_Tables_1-4 (1).docx]

**Suppl. Table 1:** Proteins showing abundance changes of more than 1.4 fold after treatment with VD3 compared to control (*higher abundant proteins*). PSM: peptide spectral matches.

| **Uniprot-ID** | **Name(s)** | **T/C** | **PSM** | **Unique peptides** | **Sequence coverage** |
| --- | --- | --- | --- | --- | --- |
| Q07973 | 1,25-dihydroxyvitamin D(3) 24-hydroxylase, mitochondrial (24-OHase) (Vitamin D(3) 24‑hydroxylase) (EC 1.14.15.16) (Cytochrome P450 24A1) (Cytochrome P450-CC24) | 3.08 | 6 | 4 | 9.92 % |
| O00338 | Sulfotransferase 1C2 (ST1C2) (EC 2.8.2.-) (Sulfotransferase 1C1) (SULT1C#1) (humSULTC2) | 2.77 | 3 | 2 | 6.08 % |
| P02452 | Collagen alpha-1(I) chain (Alpha-1 type I collagen) | 2.60 | 33 | 16 | 12.64 % |
| O15075 | Serine/threonine-protein kinase DCLK1 (EC 2.7.11.1) (Doublecortin domain-containing protein 3A) (Doublecortin-like and CAM kinase-like 1) (Doublecortin-like kinase 1) | 2.34 | 29 | 10 | 16.22 % |
| Q9H3M7 | Thioredoxin-interacting protein (Thioredoxin-binding protein 2) (Vitamin D3 up-regulated protein 1) | 1.84 | 8 | 3 | 12.02 % |
| Q9UMN6 | Histone-lysine N-methyltransferase 2B (Lysine N‑methyltransferase 2B) (EC 2.1.1.43) (Myeloid/lymphoid or mixed-lineage leukemia protein 4) (WW domain-binding protein 7) | 1.82 | 2 | 2 | 2.03 % |
| Q7Z2Z2 | Elongation factor-like GTPase 1 (Elongation factor Tu GTP-binding domain-containing protein 1) (Elongation factor-like 1) (Protein FAM42A) | 1.75 | 52 | 19 | 28.93 % |
| P08779 | Keratin, type I cytoskeletal 16 (Cytokeratin-16) (CK‑16) (Keratin-16) (K16) | 1.66 | 7 | 4 | 30.66 % |
| Q96JQ2 | Calmin (Calponin-like transmembrane domain protein) | 1.63 | 48 | 20 | 22.36 % |
| O43315 | Aquaporin-9 (AQP-9) (Aquaglyceroporin-9) (Small solute channel 1) | 1.56 | 2 | 2 | 15.59 % |
| O75363 | Breast carcinoma-amplified sequence 1 (Amplified and overexpressed in breast cancer) | 1.54 | 20 | 10 | 21.58 % |
| Q9NX52 | Rhomboid-related protein 2 (RRP2) (EC 3.4.21.105) (Rhomboid-like protein 2) | 1.53 | 6 | 3 | 12.87 % |
| P78383 | Solute carrier family 35 member B1 (UDP-galactose transporter-related protein 1) (UGTrel1) | 1.50 | 5 | 2 | 8.07 % |
| Q9UHY8 | Fasciculation and elongation protein zeta-2 (Zygin II) (Zygin-2) | 1.49 | 2 | 2 | 6.23 % |
| Q5VYY1 | Ankyrin repeat domain-containing protein 22 | 1.46 | 11 | 3 | 13.61 % |
| P48735 | Isocitrate dehydrogenase [NADP], mitochondrial (IDH) (EC 1.1.1.42) (ICD-M) (IDP) (NADP(+)-specific ICDH) (Oxalosuccinate decarboxylase) | 1.46 | 182 | 29 | 64.82 % |
| Q8N1N4 | Keratin, type II cytoskeletal 78 (Cytokeratin-78) (CK‑78) (Keratin-5b) (Keratin-78) (K78) (Type-II keratin Kb40) | 1.45 | 19 | 8 | 25.58 % |
| Q9HBU6 | Ethanolamine kinase 1 (EKI 1) (EC 2.7.1.82) | 1.43 | 6 | 3 | 9.73 % |
| P17066 | Heat shock 70 kDa protein 6 (Heat shock 70 kDa protein B') | 1.42 | 6 | 2 | 25.35 % |
| P11413 | Glucose-6-phosphate 1-dehydrogenase (G6PD) (EC 1.1.1.49) | 1.41 | 310 | 43 | 81.17 % |

**Suppl. Table 2:** Proteins showing abundance changes between 1.4 and 1.25 fold after treatment with VD3 compared to control (*moderately higher abundant proteins*). PSM: peptide spectral matches.

| **Uniprot-ID** | **Name(s)** | **T/C** | **PSM** | **Unique peptides** | **Sequence coverage** |
| --- | --- | --- | --- | --- | --- |
| Q7Z2K6 | Endoplasmic reticulum metallopeptidase 1 (EC 3.4.-.-) (Felix-ina) | 1.34 | 76 | 22 | 29.54 |
| Q6IC98 | GRAM domain-containing protein 4 (Death-inducing protein) | 1.38 | 52 | 17 | 37.02 % |
| P55061 | Bax inhibitor 1 (BI-1) (Testis-enhanced gene transcript protein) (Transmembrane BAX inhibitor motif-containing protein 6) | 1.35 | 7 | 2 | 3.80 % |
| O15061 | Synemin (Desmuslin) | 1.34 | 5 | 2 | 1.41 % |
| B0FP48 | Uroplakin-3b-like protein | 1.32 | 2 | 2 | 8.37 % |
| O14684 | Prostaglandin E synthase (EC 5.3.99.3) (Microsomal glutathione S-transferase 1-like 1) (MGST1-L1) (Microsomal prostaglandin E synthase 1) (MPGES-1) (p53-induced gene 12 protein) | 1.32 | 26 | 5 | 23.68 % |
| P19012 | Keratin, type I cytoskeletal 15 (Cytokeratin-15) (CK‑15) (Keratin-15) (K15) | 1.31 | 103 | 18 | 85.09 % |
| P47989 | Xanthine dehydrogenase/oxidase [Includes: Xanthine dehydrogenase (XD) (EC 1.17.1.4); Xanthine oxidase (XO) (EC 1.17.3.2) (Xanthine oxidoreductase) (XOR)] | 1.31 | 4 | 2 | 4.35 % |
| P04066 | Tissue alpha-L-fucosidase (EC 3.2.1.51) (Alpha‑L‑fucosidase I) (Alpha-L-fucoside fucohydrolase 1) (Alpha-L-fucosidase 1) | 1.27 | 143 | 19 | 43.35 % |
| P02533 | Keratin, type I cytoskeletal 14 (Cytokeratin-14) (CK‑14) (Keratin-14) (K14) | 1.27 | 5 | 2 | 31.36 % |
| Q9UJC5 | SH3 domain-binding glutamic acid-rich-like protein 2 (Fovea-associated SH3 domain-binding protein) | 1.26 | 20 | 5 | 59.81 % |
| P30740 | Leukocyte elastase inhibitor (LEI) (Monocyte/neutrophil elastase inhibitor) (EI) (M/NEI) (Peptidase inhibitor 2) (PI-2) (Serpin B1) | 1.26 | 353 | 40 | 75.46 % |
| P02144 | Myoglobin | 1.26 | 24 | 6 | 66.01 % |
| O94768 | Serine/threonine-protein kinase 17B (EC 2.7.11.1) (DAP kinase-related apoptosis-inducing protein kinase 2) | 1.26 | 18 | 4 | 14.25 % |
| Q6IQ23 | Pleckstrin homology domain-containing family A member 7 (PH domain-containing family A member 7) | 1.25 | 32 | 16 | 15.79 % |
| O15327 | Type II inositol 3,4-bisphosphate 4-phosphatase (EC 3.1.3.66) (Inositol polyphosphate 4-phosphatase type II) | 1.25 | 104 | 29 | 43.18 % |

**Suppl. Table 3:** Proteins showing abundance changes of lower than 0.71 fold after treatment with VD3 compared to control (*lower abundant proteins*). PSM: peptide spectral matches.

| **Uniprot-ID** | **Name(s)** | **T/C** | **PSM** | **Unique peptides** | **Sequence coverage** |
| --- | --- | --- | --- | --- | --- |
| P55087 | Aquaporin-4 (AQP-4) (Mercurial-insensitive water channel) (MIWC) (WCH4) | 0.49 | 17 | 6 | 16.72 % |
| P07988 | Pulmonary surfactant-associated protein B (SP-B) (18 kDa pulmonary-surfactant protein) (6 kDa protein) (Pulmonary surfactant-associated proteolipid SPL(Phe)) | 0.54 | 169 | 31 | 62.73 % |
| P48645 | Neuromedin-U [Cleaved into: Neuromedin-U-25 (NmU-25)] | 0.54 | 3 | 3 | 17.82 % |
| O95436 | Sodium-dependent phosphate transport protein 2B (Sodium-phosphate transport protein 2B) (Na(+)‑dependent phosphate cotransporter 2B) (NaPi3b) (Sodium/phosphate cotransporter 2B) (Na(+)/Pi cotransporter 2B) (NaPi-2b) (Solute carrier family 34 member 2) | 0.56 | 191 | 29 | 33.19 % |
| Q6ZMB0 | Acetylgalactosaminyl-O-glycosyl-glycoprotein beta-1,3-N-acetylglucosaminyltransferase (EC 2.4.1.147) (Core 3 synthase) (UDP‑GlcNAc:betaGal beta‑1,3‑N‑acetylglucosaminyl-transferase 6) (BGnT-6) (Beta-1,3-Gn-T6) (Beta-1,3-N-acetylglucosaminyltransferase 6) (Beta3Gn-T6) | 0.63 | 25 | 9 | 31.25 % |
| P05362 | Intercellular adhesion molecule 1 (ICAM-1) (Major group rhinovirus receptor) (CD antigen CD54) | 0.66 | 195 | 27 | 55.83 % |
| Q8NF37 | Lysophosphatidylcholine acyltransferase 1 (LPC acyltransferase 1) (LPCAT-1) (LysoPC acyltransferase 1) (EC 2.3.1.23) (1‑acylglycerophosphocholine O-acyltransferase) (1‑alkylglycerophosphocholine O-acetyltransferase) (EC 2.3.1.67) (Acetyl-CoA:lyso-platelet-activating factor acetyltransferase) (Acetyl-CoA:lyso-PAF acetyltransferase) (Lyso-PAF acetyltransferase) (LysoPAFAT) (Acyltransferase-like 2) (Phosphonoformate immuno-associated protein 3) | 0.69 | 176 | 28 | 53.56 % |
| Q12983 | BCL2/adenovirus E1B 19 kDa protein-interacting protein 3 | 0.71 | 15 | 3 | 17.53 % |
| Q9BY76 | Angiopoietin-related protein 4 (Angiopoietin-like protein 4) (Hepatic fibrinogen/angiopoietin-related protein) (HFARP) | 0.71 | 15 | 6 | 20.44 % |

**Suppl. Table 4:** Proteins showing abundance changes between 0.8 and 0.71 fold after treatment with VD3 compared to control (*moderately lower abundant proteins*). PSM: peptide spectral matches.

| **Uniprot-ID** | **Name(s)** | **T/C** | **PSM** | **Unique peptides** | **Sequence coverage** |
| --- | --- | --- | --- | --- | --- |
| O00622 | Protein CYR61 (CCN family member 1) (Cysteine-rich angiogenic inducer 61) (Insulin-like growth factor-binding protein 10) (IBP-10) (IGF-binding protein 10) (IGFBP-10) (Protein GIG1) | 0.71 | 52 | 17 | 48.82 % |
| O95340 | Bifunctional 3'-phosphoadenosine 5'-phosphosulfate synthase 2 (PAPS synthase 2) (PAPSS 2) (Sulfurylase kinase 2) (SK 2) (SK2) [Includes: Sulfate adenylyltransferase (EC 2.7.7.4) (ATP-sulfurylase) (Sulfate adenylate transferase) (SAT); Adenylyl-sulfate kinase (EC 2.7.1.25) (3'‑phosphoadenosine-5'-phosphosulfate synthase) (APS kinase) (Adenosine-5'-phosphosulfate 3'‑phosphotransferase) (Adenylylsulfate 3'‑phosphotransferase)] | 0.74 | 76 | 22 | 49.67 % |
| P11166 | Solute carrier family 2, facilitated glucose transporter member 1 (Glucose transporter type 1, erythrocyte/brain) (GLUT-1) (HepG2 glucose transporter) | 0.74 | 71 | 10 | 17.48 % |
| Q6UW10 | Surfactant-associated protein 2 (Surfactant-associated protein G) (SP-G) | 0.75 | 3 | 2 | 24.36 % |
| O75845 | Lathosterol oxidase (EC 1.14.19.20) (C-5 sterol desaturase) (Delta(7)-sterol 5-desaturase) (Delta(7)-sterol C5(6)-desaturase) (Lathosterol 5-desaturase) (Sterol-C5-desaturase) | 0.77 | 11 | 4 | 12.71 % |
| Q96C24 | Synaptotagmin-like protein 4 (Exophilin-2) (Granuphilin) | 0.77 | 2 | 2 | 4.32 % |
| Q9ULC5 | Long-chain-fatty-acid--CoA ligase 5 (EC 6.2.1.3) (Long-chain acyl-CoA synthetase 5) (LACS 5) | 0.78 | 202 | 34 | 59.88 % |
| P55011 | Solute carrier family 12 member 2 (Basolateral Na‑K‑Cl symporter) (Bumetanide-sensitive sodium-(potassium)-chloride cotransporter 1) | 0.79 | 63 | 24 | 23.43 % |
| O15347 | High mobility group protein B3 (High mobility group protein 2a) (HMG-2a) (High mobility group protein 4) (HMG-4) | 0.79 | 326 | 29 | 64.50 % |
| Q08357 | Sodium-dependent phosphate transporter 2 (Gibbon ape leukemia virus receptor 2) (GLVR-2) (Phosphate transporter 2) (PiT-2) (Pit2) (hPit2) (Solute carrier family 20 member 2) | 0.79 | 13 | 6 | 12.27 % |
| P22532 | Small proline-rich protein 2D (SPR-2D) (Small proline-rich protein II) (SPR-II) | 0.80 | 39 | 6 | 80.56 % |
