# Supplementary figures and images for: Alveolar–Capillary Barrier Protection In Vitro: Lung Cell Type-Specific Effects and Molecular Mechanisms Induced by 1α, 25-Dihydroxyvitamin D3
Source: Int J Mol Sci. 2023 Apr 14;24(8):7298. doi: 10.3390/ijms24087298 (PMC10138495; doi:10.3390/ijms24087298)

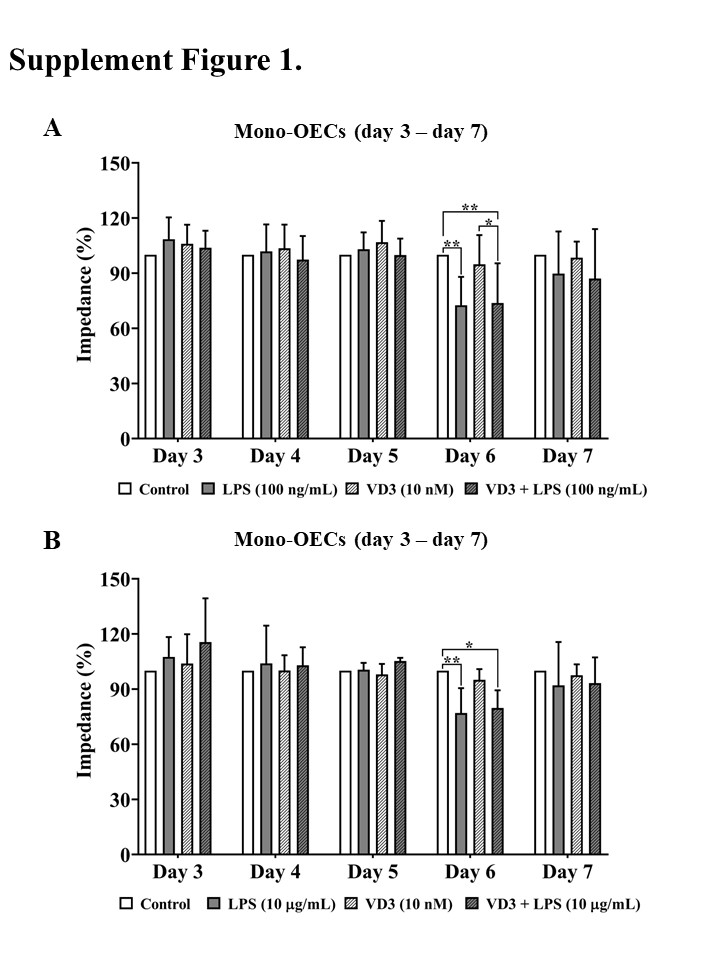

Supplement: Supplementary file 1 [file ijms-24-07298-s001.zip › ijms-2319618-supplementary/Supplemental_proofs/Suppl. Fig 1.jpg]

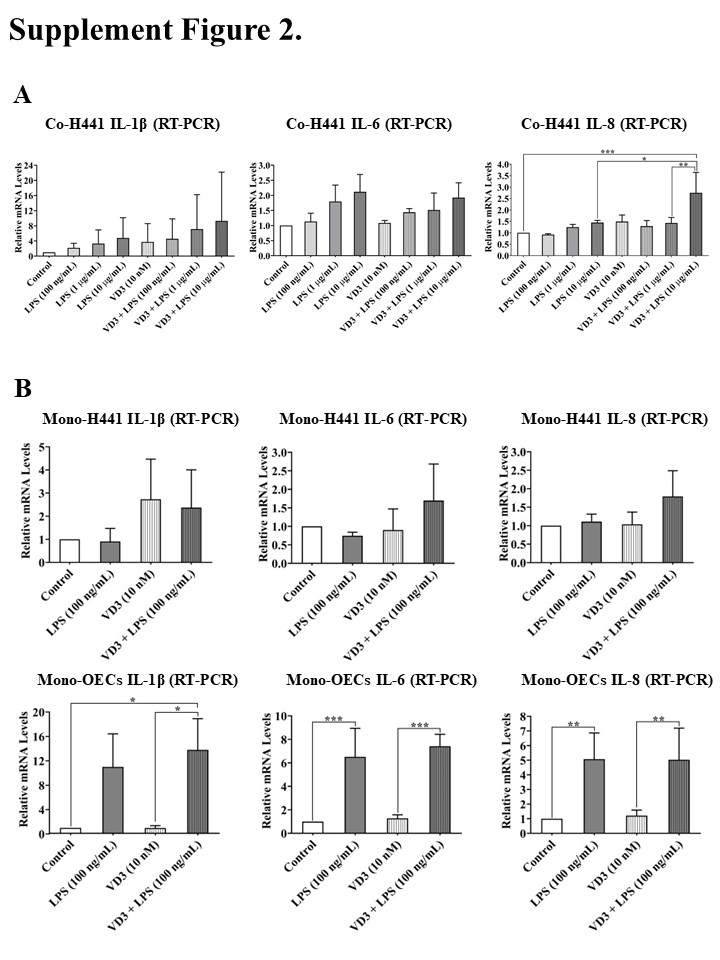

Supplement: Supplementary file 1 [file ijms-24-07298-s001.zip › ijms-2319618-supplementary/Supplemental_proofs/Suppl. Fig 2.jpg]
